# Evolution of Key Oxygen-Sensing Genes Is Associated with Hypoxia Tolerance in Fishes

**DOI:** 10.1093/gbe/evae183

**Published:** 2024-08-21

**Authors:** Courtney H Babin, Félix P Leiva, Wilco C E P Verberk, Bernard B Rees

**Affiliations:** Department of Biological Sciences, University of New Orleans, New Orleans, LA 70148, USA; Alfred Wegener Institute, Helmholtz Centre for Polar and Marine Research, Bremerhaven 27570, Germany; Department of Animal Ecology and Physiology, Radboud University Nijmegen, Nijmegen, The Netherlands; Department of Biological Sciences, University of New Orleans, New Orleans, LA 70148, USA

**Keywords:** hypoxia, critical oxygen tension, Actinopterygii, positive selection, hypoxia-inducible factor alpha, prolyl hydroxylase domain

## Abstract

Low dissolved oxygen (hypoxia) is recognized as a major threat to aquatic ecosystems worldwide. Because oxygen is paramount for the energy metabolism of animals, understanding the functional and genetic drivers of whole-animal hypoxia tolerance is critical to predicting the impacts of aquatic hypoxia. In this study, we investigate the molecular evolution of key genes involved in the detection of and response to hypoxia in ray-finned fishes: the prolyl hydroxylase domain (*PHD*)–hypoxia-inducible factor (*HIF*) oxygen-sensing system, also known as the *EGLN* (egg-laying nine)–*HIF* oxygen-sensing system. We searched fish genomes for *HIFA* and *EGLN* genes, discovered new paralogs from both gene families, and analyzed protein-coding sites under positive selection. The physicochemical properties of these positively selected amino acid sites were summarized using linear discriminants for each gene. We employed phylogenetic generalized least squares to assess the relationship between these linear discriminants for each *HIFA* and *EGLN* and hypoxia tolerance as reflected by the critical oxygen tension (*P*_crit_) of the corresponding species. Our results demonstrate that *P*_crit_ in ray-finned fishes correlates with the physicochemical variation of positively selected sites in specific *HIFA* and *EGLN* genes. For *HIF2A*, two linear discriminants captured more than 90% of the physicochemical variation of these sites and explained between 20% and 39% of the variation in *P*_crit_. Thus, variation in *HIF2A* among fishes may contribute to their capacity to cope with aquatic hypoxia, similar to its proposed role in conferring tolerance to high-altitude hypoxia in certain lineages of terrestrial vertebrates.

**Significance**

Decreased oxygen availability (hypoxia) poses a threat to animal life, especially in aquatic habitats where human activities have increased the duration, severity, and geographic extent of hypoxia. This surge in aquatic hypoxia is expected to unevenly impact the distribution of aquatic species, including ray-finned fishes, the most widespread and speciose group of vertebrates. This study investigates whether the hypoxia tolerance of ray-finned fishes, defined by the oxygen partial pressure threshold for sustaining aerobic metabolism, is linked to sequence variation in critical oxygen-sensing genes. The findings reveal that physicochemical variation in a key transcription factor is associated with variation in this measure of hypoxia tolerance of adult fish among the ray-finned fishes.

## Introduction

Oxygen is essential for the development, growth, reproduction, and survival of contemporary life on Earth. Hence, a decrease in oxygen levels below fully air-saturated conditions (hypoxia) can be stressful, especially for water-breathing animals due to the much lower concentration and diffusion rates of oxygen in water compared to air (Nikinmaa and Rees 2005; Verberk et al. 2011). Although hypoxia occurs naturally in some aquatic habitats (Diaz and Breitburg 2009; Mandic and Regan 2018; Woods et al. 2022), the last several decades have seen a dramatic increase in the severity, duration, and geographic scope of aquatic hypoxia due to human activities (Breitburg et al. 2018; Sampaio et al. 2021; Xenopoulos et al. 2021). Higher water temperatures due to global warming decrease oxygen solubility, increase biological oxygen consumption rates, and intensify vertical stratification of the water column, thereby limiting the mixing of well-aerated surface waters with deeper oxygen-poor water. Nutrient runoff due to altered land use further contributes to oxygen depletion by stimulating the growth of nutrient-limited microorganisms. Thus, aquatic deoxygenation is recognized as an increasing threat to marine and freshwater ecosystems worldwide (Diaz and Rosenberg 2008; Breitburg et al. 2018; Jane et al. 2021).

The central molecular pathway regulating the cellular responses of animals to hypoxia includes the hypoxia-inducible factor (HIF) family of transcription factors and the prolyl hydroxylase domain (PHD) enzymes (Fong and Takeda 2008; Kaelin and Ratcliffe 2008; Semenza 2009; Ivan and Kaelin 2017). HIF is a family of heterodimeric transcription factors, with the functional transcription factor consisting of an oxygen-sensitive alpha subunit (HIFA) and a constitutively expressed beta subunit, also known as the aryl hydrocarbon receptor nuclear translocator (ARNT). The PHD enzymes catalyze the oxygen-dependent hydroxylation of specific proline residues of HIFA subunits, a modification that targets these subunits for ubiquitination and proteasomal degradation (Maxwell et al. 1999; Bruick and McKnight 2001; Epstein et al. 2001; Ivan et al. 2001; Jaakkola et al. 2001). The genes encoding PHD are also known as *EGLN* due to their homology with the egg-laying nine gene of the nematode *Caenorhabditis elegans* (Epstein et al. 2001). A drop in cellular levels of oxygen reduces rates of HIFA hydroxylation and degradation, allowing HIFA subunits to accumulate, bind to ARNT, and regulate the expression of hundreds of genes, many of which serve to improve oxygen transport to tissues or increase the capacity of cells to survive low oxygen levels (Wenger et al. 2005; Kaelin and Ratcliffe 2008; Semenza 2011, 2012).

Vertebrate animals have multiple copies of *HIFA* and *EGLN* genes (paralogs) arising from two rounds of genome duplication at the base of vertebrate evolution (Ohno 1970; Dehal and Boore 2005; Sacerdot et al. 2018). Most terrestrial vertebrates have three paralogs of *HIFA* (*HIF1A*, *HIF2A*, and *HIF3A*), which differ in their tissue distribution and target-gene specificity (Patel and Simon 2008; Keith et al. 2012; Duan 2016; Graham and Presnell 2017), and three paralogs of *EGLN* (*EGLN1*, *EGLN2*, and *EGLN3*), which vary in their affinity for HIFA subunits (Fong and Takeda 2008; Ivan and Kaelin 2017). The diversity of *HIFA* and *EGLN* is greater among ray-finned fishes (Actinopterygii) because of additional genome duplication events during their evolution, including the teleost-specific genome duplication (TGD, 350 to 320 Mya; Postlethwait et al. 2000; Hoegg et al. 2004; Volff 2005; Amores et al. 2011), and additional rounds of genome duplication in lineages leading to carp (19.5 Mya; Zhang et al. 2019; Wang et al. 2022), goldfish (12 to 10 Mya; Farhat et al. 2022; Wang et al. 2022), and salmonids (100 to 25 Mya; Berthelot et al. 2014; Macqueen and Johnston 2014). Phylogenetic analyses of *HIFA* in ray-finned fishes showed that many lineages retain the four *HIFA* genes predicted from two rounds of genome duplication in the ancestor to all vertebrates, with additional teleost-specific paralogs of *HIF1A* and *HIF2A* retained in the selected lineages (Rytkonen et al. 2013; Mandic et al. 2021; Townley et al. 2022). Moreover, evidence has been presented for additional *HIFA* paralogs arising from the genome duplication in lineages leading to carp (Zhang et al. 2019) and salmonids (Townley et al. 2022). Rytkonen et al. (2011) suggested that fishes have three *EGLN* genes that are homologous to those seen in terrestrial vertebrates, and more recently, Farhat et al. (2022) provided evidence of lineage-specific duplicates in goldfish.

Given this tremendous diversity in *HIFA* and *EGLN* among fishes, combined with the fact that Actinopterygii are the most speciose group of vertebrates and occur in aquatic habitats spanning a range of oxygen concentrations (Nelson et al. 2016), we hypothesized that sequence variation in *HIFA* and *EGLN* is related to variation in hypoxia tolerance among fishes. One measure of hypoxia tolerance is the critical oxygen tension (*P*_crit_), the lowest level of ambient oxygen at which the energetic costs of maintenance can be met by aerobic metabolism (Ultsch et al. 1981; Farrell and Richards 2009; Claireaux and Chabot 2016; Reemeyer and Rees 2019). At oxygen tensions below *P*_crit_, anaerobic processes are recruited or metabolism is suppressed. Because neither strategy can be maintained indefinitely, survival ultimately decreases at oxygen levels below *P*_crit_ (Farrell and Richards 2009). It should be noted that the capacity to tolerate hypoxia is a complex phenotype, the utility of *P*_crit_ has been questioned (Wood 2018), and alternative metrics of hypoxia tolerance of fishes exist (Alexander and McMahon 2004; Rees and Matute 2018; Seibel et al 2021). Nevertheless, *P*_crit_ remains an ecologically and physiologically relevant index of the capacity for fish to extract oxygen from their surroundings (Regan et al. 2019), and it has been determined for a broader range of fishes than other measures of hypoxia tolerance (Rogers et al. 2016; Verberk et al. 2022a).

In the present study, therefore, we investigated whether genomic variation in the coding sequences (CDSs) of *HIFA* and *EGLN* is related to variation in the hypoxia tolerance of ray-finned fishes, as reflected by their *P*_crit_. Phylogenetic relationships were reconstructed for all *HIFA* and *EGLN* paralogs from currently available Actinopterygian genomes. Reasoning that positive selection could contribute to divergence within and among paralogs (Liao and Zhang 2006; Lian et al. 2020), we tested each gene for positive selection and grouped the *HIFA* and *EGLN* genes from different species according to the physicochemical properties of amino acid sites putatively under positive selection. While this analytical framework is similar to the recent analyses by Townley et al. (2022), the current study used more species, including 16 not represented in Townley et al. (2022), and a larger collection of paralogs, with over 200 new or updated sequences. The central and novel aspect of the current study, however, is that we took advantage of a comprehensive comparative analysis of *P*_crit_ among fishes (Verberk et al. 2022a) and assessed the relationships between the physicochemical properties of *HIFA* and *EGLN* and the *P*_crit_ values for the corresponding species. Prior to correcting for phylogeny, we found that physicochemical variation in *HIFA* and *EGLN* was significantly related to variation in *P*_crit_. After correcting for phylogeny, sequence variation in *HIF1A* and *HIF2A* was associated with variation in *P*_crit_, with variation in *HIF2A* being more strongly related to variation in *P*_crit_ than other oxygen-sensing genes in this sample of ray-finned fishes.

## Results and Discussion

### Species Used for Analysis

**Fig. 1.**
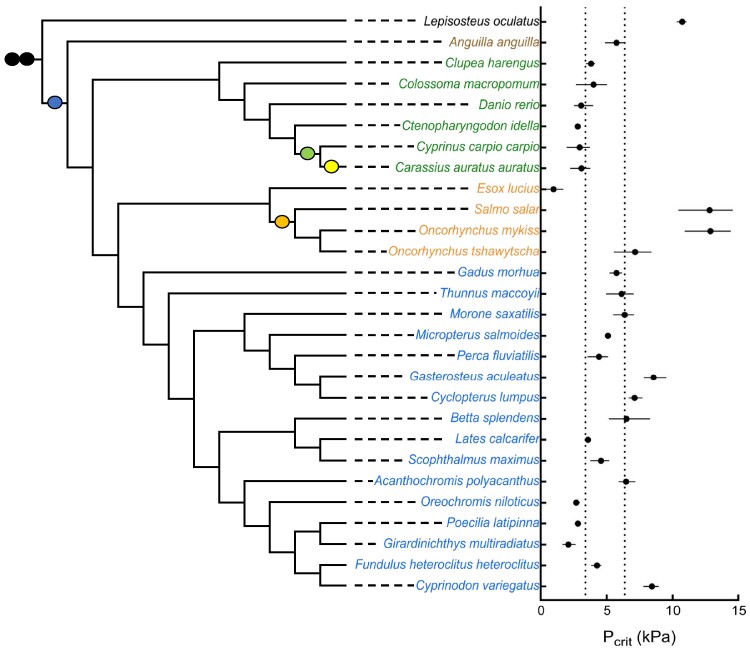
The phylogenetic relationships and hypoxia tolerance of the 28 species of Actinopterygii included in this study. The phylogeny was synthesized with the Open Tree of Life (opentreeoflife.org). Filled circles indicate genome duplication events at the base of vertebrate evolution (black) and during the evolution of teleost fishes (blue), carp (green), goldfish (yellow), and salmonids (orange). Species are color coded according to their phylogenetic placement: *L. oculatus* (spotted gar), representing a basal ray-finned fish that diverged prior to the TGD, black; the basal teleost *A. anguilla* (European eel), brown; Otocephala (Atlantic herring, tambaqui, zebrafish, grass carp, common carp, and goldfish), green; Salmonidae and their sister group (*E. lucius*, northern pike), orange; and Neoteleostei (more-derived teleosts), blue. On the right are values of standardized critical oxygen tensions (*P*_crit_ in kPa), represented as dots (median) and bars (range) of values determined at three temperatures (15, 24, and 28 °C) for each species (see Materials and Methods). The vertical lines represent the lower (left) and upper (right) 20th percentile of the *P*_crit_ values based on all ray-finned fish species in Verberk et al. (2022a) plus spotted gar, Atlantic herring, and northern pike. For comparison, the oxygen tension of air-saturated water is 21 kPa.

This study included all species of ray-finned fishes for which sequenced genomes and critical oxygen tension (*P*_crit_) were available (Fig. 1). This collection of 28 species represents 14 orders of Actinopterygii spanning over 300 million years of evolution. This sample of ray-finned fishes included the spotted gar (*Lepisosteus oculatus*) that diverged approximately 320 Mya from other ray-finned fishes, i.e. prior to the TGD (Hoegg et al. 2004; Amores et al. 2011; Braasch et al. 2016; but see Davesne et al. 2021). Other species arose after the TGD and represent most major lineages of ray-finned fishes, including some that experienced additional lineage-specific genome or gene duplication events (Otocephala and Salmoniformes). These species exhibit a range of tolerance to reductions in ambient oxygen from very tolerant (low *P*_crit_) to very sensitive (high *P*_crit_).

### Phylogenetic Relationships of Actinopterygian *HIFA* and *EGLN*

**Fig. 2.**
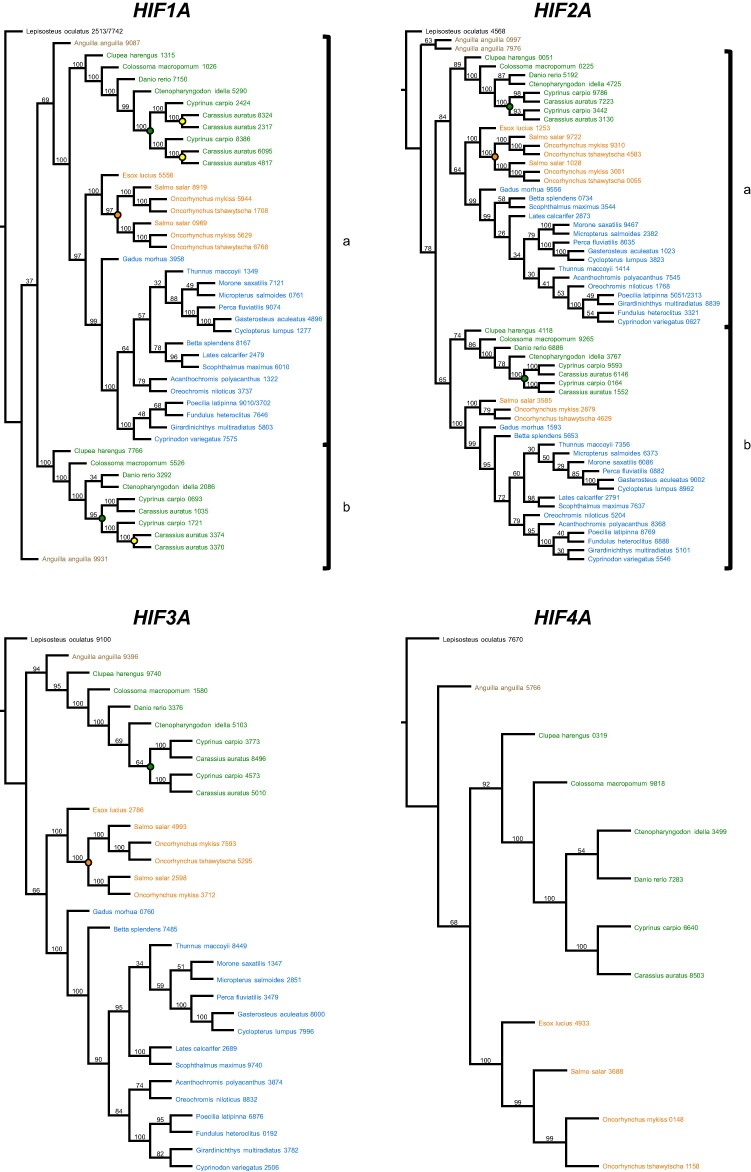
ML phylogenetic inferences of Actinopterygian *HIFA* genes using CDSs. Evolutionary analyses were conducted in RAxML v 8.2.11 using the general time reversible substitution matrix (GAMMA+P-Invar model). The most likely tree inferred from 100 bootstrap replicates is shown for each gene with bootstrap values above the corresponding branches. Taxa are color coded as in Fig. 1. Where applicable, teleost-specific gene duplications are identified as “a” and “b” next to brackets. Genome duplications are indicated by filled circles: common carp-specific (green), goldfish-specific (yellow), and salmonid-specific (orange). Sequences are identified by the species names followed by the last 4 digits of the NCBI or Ensembl reference gene accession numbers (see supplementary table S1, Supplementary Material online, for a full list of genes).

**Fig. 3.**
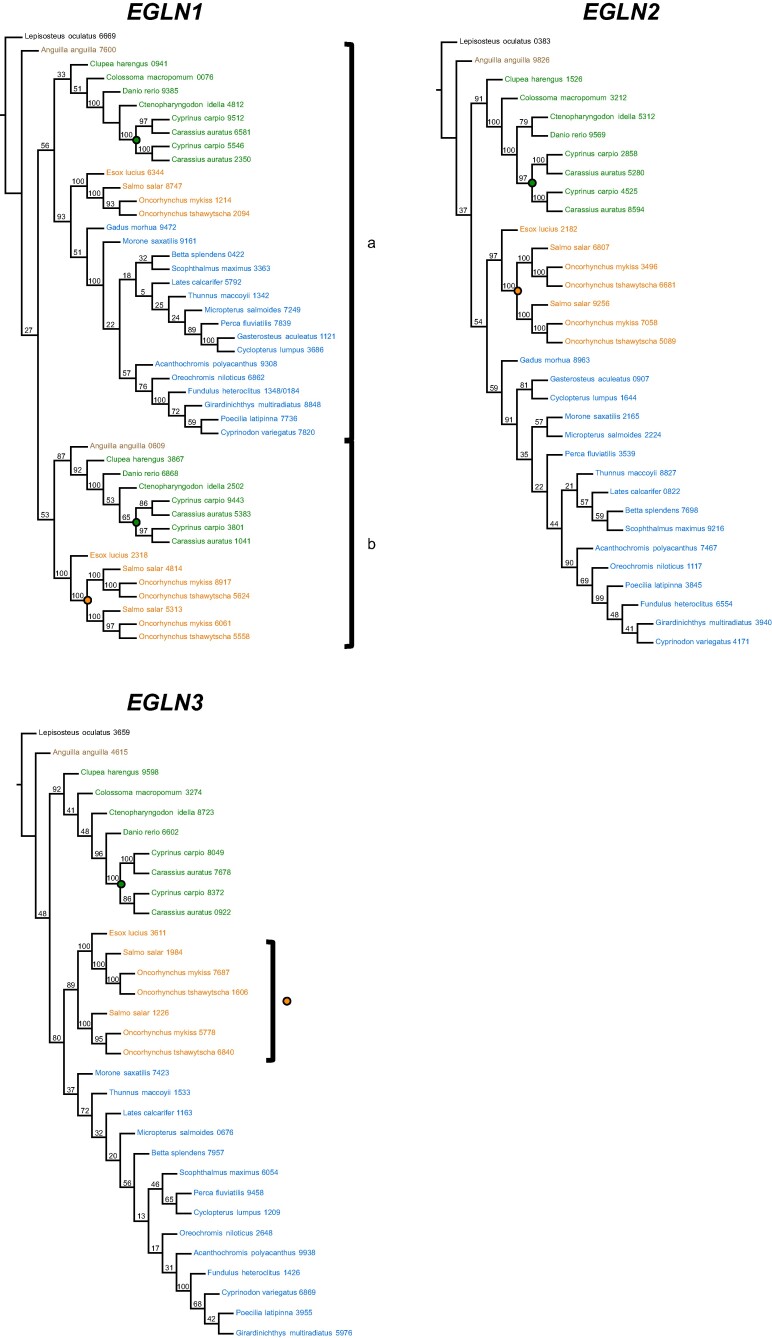
ML phylogenetic inferences of Actinopterygian *EGLN* genes using CDSs. Evolutionary analyses were conducted in RAxML v 8.2.11 using the general time reversible substitution matrix (GAMMA+P-Invar model). The most likely tree inferred from 100 bootstrap replicates is shown for each gene with bootstrap values above the corresponding branches. Taxa are color coded as in Fig. 1. Where applicable, teleost-specific gene duplications are identified as “a” and “b” next to brackets. Genome duplications are indicated by filled circles: common carp-specific (green), goldfish-specific (yellow), and salmonid-specific (orange). Sequences are identified by the species names followed by the last 4 digits of the NCBI or Ensembl reference gene accession numbers (see supplementary table S1, Supplementary Material online, for a full list of genes).

Searching the genomes of these species resulted in 150 *HIFA* homologs and 109 *EGLN* homologs (supplementary table S1, Supplementary Material online). Phylogenetic analyses resolved four *HIFA* (Fig. 2) and three *EGLN* (Fig. 3) homology groups, largely congruent with earlier studies (Rytkonen et al. 2011, 2013; Li et al. 2022; Townley et al. 2022; Yu et al. 2023). Across all genes, the placement of species was essentially identical to the fossil-calibrated phylogeny of ray-finned fishes (Hughes et al. 2018). Importantly, our search did not recover *HIF4A* in Neoteleostei (Fig. 2), nor a fourth *EGLN* in any ray-finned fish (Fig. 3), suggesting that these were lost during (*HIF4A*) or prior to (*EGLN4*) the evolution of ray-finned fishes.

Spotted gar possesses only one paralog each of *HIF1A-4A* and *EGLN1-3* as expected from its early divergence from the rest of ray-finned fishes. The remaining species investigated here occur in lineages that diverged after the TGD, resulting in teleost-specific paralogs, “a” and “b,” of each gene; however, two teleost-specific paralogs were only recovered for *HIF1A*, *HIF2A*, and *EGLN1*, and these were retained in only certain lineages. For *HIF1A* (Fig. 2), all searched species retained *HIF1Aa*, inferred to be more closely related to ancestral *HIF1A* by similarity of flanking genes (Gasanov et al. 2021; Townley et al. 2022), whereas only European eel (*Anguilla anguilla*) and Otocephala (including herring, tambaqui, zebrafish, carp, and goldfish) retained *HIF1Ab*. These results suggest that *HIF1Ab* was lost in the lineage leading to Neoteleostei after the divergence of Otocephala (228 Mya; Benton et al. 2015). On the other hand, most species have both teleost-specific paralogs of *HIF2A*. One form encodes a full-length protein in all species included here (supplementary table S1, Supplementary Material online). This paralog was the first *HIF2A* described in fishes (Powell and Hahn 2002) and shares more flanking genes with the ancestral *HIF2A*, represented by spotted gar (Townley et al. 2022). For these reasons, it is referred to as *HIF2Aa* (Rytkonen et al. 2013; Townley et al. 2022), although it has been designated as *EPAS1b* or *HIF2Ab* in many databases (supplementary table S1, Supplementary Material online). In contrast, the other paralog, herein referred to as *HIF2Ab*, encodes a full-length protein only in European eel and Otocephala and a truncated protein in Salmonidae (and their sister taxa *Esox lucius*) and Neoteleostei (supplementary table S1, Supplementary Material online; Rytkonen et al. 2013; Townley et al. 2022). *EGLN1a* was recovered in all taxa, but *EGLN1b* was present only in less-derived species (*A. anguilla*, Otocephala, *E. lucius*, Salmonidae) and absent in the Neotelestei (Fig. 3), consistent with the loss of this paralog after the divergence of Salmonidae (100 to 70 Mya; Shedko et al. 2013). The current analysis did not recover two paralogs of *HIF3A*, *HIF4A*, *EGLN2*, and *EGLN3* in any species, suggesting that one form of each was nonfunctionalized shortly after the TGD, a common fate for gene duplicates (Lynch and Conery 2000).

Additional *HIFA* and *EGLN* paralogs were recovered in carp, goldfish, and salmonids, presumably resulting from lineage-specific genome or gene duplication events (Berthelot et al. 2014; Macqueen and Johnston 2014; Kuang et al. 2016; Zhang et al. 2019; Farhat et al. 2022; Wang et al. 2022). Carp-specific paralogs were recovered for *HIF1Aa*, *HIF1Ab*, *HIF2Aa*, *HIF2Ab*, and *HIF3A*, with additional goldfish-specific paralogs for *HIF1Aa* and *HIF1Ab* (green and yellow nodes, respectively, in Fig. 2), resulting in a minimum of 11 copies of various *HIFA* genes in common carp (*Cyprinus carpio*) and 14 copies in goldfish (*Carassius auratus*) (supplementary table S1, Supplementary Material online). Salmonid-specific duplicates of *HIF1Aa*, *HIF2Aa*, and *HIF3A* (orange nodes in Fig. 2) resulted in a total of eight copies of the various *HIFA* genes when including *HIF2Ab* and *HIF4A* (supplementary table S1, Supplementary Material online). Within the *EGLN* homology group, carp-specific duplicates were recovered for *EGLN1a*, *EGLN1b*, *EGLN2*, and *EGLN3*, and salmonid-specific duplicates were recovered for all *EGLN* genes except *EGLN1a* (green and orange nodes, respectively, in Fig. 3; supplementary table S1, Supplementary Material online). Many of these lineage-specific *HIFA* and *EGLN* paralogs have not been reported previously, and the elucidation of their expression and functions will require experiments capable of differentiating them (e.g. with paralog-specific probes).

### Evidence of Positive Selection in the Evolution of Actinopterygian *HIFA* and *EGLN*

Reasoning that positive selection may have contributed to the diversity of *HIFA* and *EGLN* among fishes, we tested for positive selection using codon-based mixed effects models for episodic selection (mixed effects model of evolution [MEME]; Murrell et al. 2012) and gene-wide Bayesian tests of episodic diversification (branch-site unrestricted statistical test for episodic diversification [BUSTED]; Murrell et al. 2015). MEME detected episodic positive selection in all homology groups, with the number of sites putatively experiencing episodic positive selection ranging from eight for *EGLN1* to 53 for *HIF2A* (Table 1). Most of these sites (>75%) also had BUSTED evidence ratios greater than 2, providing additional support for episodic positive selection occurring at these sites. Pervasive positive selection was evaluated with fixed effects likelihoods (FEL) for pervasive selection (Kosakovsky Pond and Frost 2005b) and found to occur much less frequently than episodic selection in all *HIFA* and not at all for *EGLN* genes (Table 1). This is consistent with the notion that episodic positive selection occurs at more sites than pervasive selection across a wide range of organisms (Murrell et al. 2012), potentially due to greater selective pressures in those lineages that experience more variable environments.

**Table 1:** Codons putatively under positive selection in *HIFA* and *EGLN* genes in ray-finned fishes

Gene	Episodic selection	Pervasive selection
*HIF1A*	**97**, **140**, **142**, **183**, ***274***, **295**, **315**, *354*, **469**, **483**, 524, ***629***, 630, **642**, **656**, **660**, **735**, ***805***, **810**, **872**, **883**, ***894***, **897**, **905**, **921**, **923**, **956**, **976**	543
*HIF2A*	109, 156^a^, 199, **200**, **201**, **204**, 268^a^, **281**, *285*, **288**, **545**, ***547***, **611**, **613**, **634**, **651**, **681**^a^, **683**, **684**, **686**, ***707***, **708**, **712**, **715**, **717**, **722**, **724**, 727, ***731***, 750, **761**, **773**, **786**, **800**, **816**, **887**, 888^a^, **912**, **929**, 953, 1009, **1079**, **1085**, **1088**, **1090**, 1107, *1118*^a^, **1121**, **1163**, **1165**, **1166**, **1167**, **1234**	156^a^, 240, 268^a^, **681**^a^, 888^a^, *1118*^a^
*HIF3A*	***11*** ^a^, **25**, **89**, **176**, 311, **456**, **461**, **462**, **483**^a^, **485**, **491**, **516**^a^, **525**, **577**, **597**, **599**^a^, **602**, **610**, **643**, ***658***, ***670***, ***675***, **688**, ***720***, **832**	***11*** ^a^, 473, **483**^a^, **516**^a^, **599**^a^
*HIF4A*	**71**, **160**, **303**, **310**, *394*^a^, **546**, **561**, 715^a^, **779**^a^, 807, 854, 867, **879**, **892**^a^	75, *394*^a^, 715^a^, **779**^a^, **892**^a^
*EGLN1*	**13**, **78**, **79**, **89**, **90**, **91**, **167**, **395**	NA^b^
*EGLN2*	21, 45, 107, 245, 289, 314, 316, **335**, 379, 442, **489**, **281**, 593, **684**, **687**, **693**, **695**, **696**, **697**, **701**	NA^b^
*EGLN3*	29, 211, 223, 336, 376, 420, **461**, **469**, **476**, **477**	NA^b^

Episodic positive selection was detected by MEME, and pervasive positive selection was detected by FEL. Codon numbers correspond to the positions in the multiple sequence alignment of all *HIFA* and *EGLN* genes used in each analysis. Codons with episodic positive selection supported by BUSTED evidence ratio > 2 are shown in bold type. Codons found to be under positive selection in Townley et al. (2022) are shown in italics. See supplementary tables S2 to S8, Supplementary Material online, for amino acid identities for each *HIFA* and *EGLN* gene.

^a^Positively selected codons detected by both MEME and FEL. ^b^NA, not applicable: no sites detected by FEL.

Two observations can be made about the number of sites experiencing positive selection in *HIFA* and *EGLN* (Table 1). First, the number of sites is larger for the *HIFA* genes compared to *EGLN* genes, even after accounting for the longer CDSs for *HIFA* genes. Second, the number of codons under positive selection expressed as a proportion of the total number of codons was significantly greater in *HIF2A* than other *HIFA* genes (*P* < 0.05; χ^2^ test). Both observations could reflect a larger role for positive selection in the evolution in *HIFA*, *HIF2A* in particular, than for *EGLN* genes. However, we cannot exclude the possibility that those larger numbers of sites have experienced weaker positive selection, as compared to stronger positive selection acting on fewer sites in other genes.

Of note, many of the positively selected sites found here occurred in conserved structural or functional domains. Sites under positive selection in each *HIFA* homology group occurred in domains involved in DNA binding, protein dimerization, oxygen-dependent degradation, or activation of gene expression (supplementary tables S2 to S5, Supplementary Material online), while several sites under positive selection in the *EGLN* genes were mapped to the active site (supplementary tables S6 to S8, Supplementary Material online). In addition, for *HIF2A*, some of the positively selected sites aligned with sites known to be subject to posttranslational modification in mammals (supplementary table S3, Supplementary Material online; Albanese et al. 2020; Daly et al. 2021).

Using similar approaches, Townley et al. (2022) recently measured the prevalence of positive selection on a smaller set of fish *HIFA* genes, including some paralogs from different species. The current results agree with Townley et al. (2022) in two important ways: *HIF2A* was found to have more positively selected sites than other *HIFA* homologs and positive selection in all *HIFA* genes was found at sites predicted to be critical for protein structure and function. The specific codons identified in these two studies, however, were largely different. For *HIF1A*, *HIF2A*, and *HIF3A*, only five codons from each gene were shared between the current study and Townley et al. (2022), and for *HIF4A*, only one site was common (Table 1, italicized codons). Tests of positive selection are known to be sensitive to the number, quality, and alignment of sequences (Wong et al. 2008; Murrell et al. 2012) indicating that caution should be exercised when ascribing functional importance to any specific site identified by these tests.

Nevertheless, the current results and those of Townley et al. (2022) complement studies reporting signatures of positive selection in the *EGLN–HIF* pathway during adaptation to hypoxia associated with high altitude. Targets of positive selection include *HIF2A* and *EGLN1* in Tibetan human populations (Beall et al. 2010; Eichstaedt et al. 2017; Pamenter et al. 2020); *HIF2A* in the Tibetan mastiff (Li et al. 2014); *HIF2A* in high-altitude deer mice (Schweizer et al. 2019); and *HIF1A* and *HIF2A* in fishes from the Tibetan plateau (Guan et al. 2014; Wang et al. 2015a, b). Thus, it appears that positive selection on *HIFA* and *EGLN* has occurred among aquatic and terrestrial vertebrate lineages living in high-altitude habitats characterized by reductions in oxygen availability, potentially contributing to the hypoxia tolerance of certain species.

### *HIFA* and *EGLN* Homologs Group According to Properties of Positively Selected Sites

To better understand the potential physicochemical consequences of this positive selection, we distinguished groups of *HIFA* and *EGLN* homologs by the characteristics of amino acids putatively under positive selection using discriminant analysis of principal components (DAPC). This is a novel application of a method often employed to evaluate genetic diversity and population structure based on single nucleotide polymorphisms (SNPs) and microsatellites (Jombart et al. 2010; Dotti do Prado et al. 2017; Kajungiro et al. 2019; Sunde et al. 2020). In brief, variation in the physicochemical properties among amino acids occurring at each positively selected site for a given *HIFA* or *EGLN* homology group was summarized by principal components, followed by clustering groups of homologs (DAPC groups) along orthogonal axes of variation, or linear discriminants (LDs). Depending upon the gene, two to four DAPC groups were distinguished by their scores along one to three LDs (Table 2).

**Table 2:** Number of gene groups identified by DAPC of the physicochemical properties of positively selected sites in *HIFA* and *EGLN* in ray-finned fishes

Homolog	DAPC groups	LD1	LD2	LD3
*HIF1A*	4	0.62	0.24	0.14
*HIF2A*	4	0.80	0.14	0.06
*HIF3A*	3	0.57	0.43	NA
*HIF4A*	2	1.00	NA	NA
*EGLN1*	4	0.56	0.42	0.02
*EGLN2*	2	0.77	0.23	NA
*EGLN3*	2	1.00	NA	NA

DAPC groups were distinguished by their scores on one to three LDs, and the proportion of physicochemical variation explained by each LD is shown. See supplementary tables S2 to S8, Supplementary Material online, for specific genes in each DAPC group and codons that heavily weighted on each LD.

The species and homologs making up the DAPC groups differed for each gene (supplementary figs. S1 to S7, Supplementary Material online). As expected, phylogeny was a main contributor to the homolog groupings, e.g. segregating homologs from Otocephala, Salmonidae, and Neoteleostei for *HIF1A* (supplementary fig. S1, Supplementary Material online), *HIF3A* (supplementary fig. S3, Supplementary Material online), *EGLN1* (supplementary fig. S5, Supplementary Material online), and *EGLN2* (supplementary fig. S6, Supplementary Material online). Among Otocephala, the cyprinids (zebrafish, *Danio rerio*; grass carp, *Ctenopharyngodon idella*; common carp, *Cyprinus carpio*; goldfish, *Carassius auratus*) frequently grouped together and apart from other Otocephala (Atlantic herring, *Clupea harengus*; tambaqui, *Colossoma macropomum*), which grouped with various taxa depending upon the gene analyzed. This analysis also discriminated between teleost-specific paralogs of *HIF2A* and *EGLN1* (supplementary figs. S2 and S5, Supplementary Material online, respectively). The codons that loaded most heavily on each LD for each gene were determined (supplementary figs. S1 to S7, Supplementary Material online), along with the amino acid residues at these sites in all genes (supplementary tables S2 to S8, Supplementary Material online).

### Relating Physicochemical Variation of *HIFA* and *EGLN* to Hypoxia Tolerance

While Townley et al. (2022) used similar approaches to distinguish among a different set of *HIFA* paralogs in fishes, the current study is novel in determining if physicochemical variation of either *HIFA* or *EGLN* is related to the hypoxia tolerance of fishes. We used phylogenetic generalized least squares (PGLS) to assess the relationship between the LD scores of each *HIFA* and *EGLN* homolog and the standardized *P*_crit_ values from the corresponding species. These analyses were performed for standardized *P*_crit_ values determined at three temperatures, 15, 24, and 28 °C. Moreover, these analyses were done excluding the influence of phylogeny (λ = 0.001), as well as after accounting for the relationships among the paralogs in a given analysis (using model-selected values of λ).

Tables 3 and 4 present the best PGLS models describing variation in *P*_crit_ at 24 °C excluding and including the influence of phylogeny, respectively. Without accounting for phylogeny, variation in *P*_crit_ was found to be associated with physicochemical variation for all genes except *HIF4A* and *EGLN3* as shown by the retention of one or more LD in the best models (Table 3). Although the goodness of fit (*R*^2^) and the significance of individual LDs (*P* values) differed somewhat for *P*_crit_ at 15 °C (supplementary table S9, Supplementary Material online) and *P*_crit_ at 28 °C (supplementary table S10, Supplementary Material online), the general results were largely congruent across temperatures. When phylogeny was taken into account, *P*_crit_ was found to be associated with physicochemical variation in *HIF1A* and *HIF2A* (Table 4), which, again, was consistent across temperatures (supplementary table S11, Supplementary Material online, for *P*_crit_ at 15 °C; supplementary table S12, Supplementary Material online, for *P*_crit_ at 28 °C). After accounting for phylogeny, PGLS models relating variation in *HIF3A*, *EGLN1*, or *EGLN2* to *P*_crit_ were not significant at any temperature.

**Table 3:** Relationships between the physicochemical properties of positively selected amino acid sites in Actinopterygian *HIFA* and *EGLN* and critical oxygen tension (*P*_crit_) at 24 °C, without accounting for phylogeny

Effect	Estimate	SE	*t*	*P*
*HIF1A* (λ = 0.001; *R*^2^ = 0.480)				
(Intercept)	5.581	0.407	13.727	<0.001
LD2	0.084	0.091	0.917	0.364
LD3	0.635	0.113	5.632	<0.001
*HIF2A* (λ = 0.001; *R*^2^ = 0.507)				
(Intercept)	5.952	0.462	7.603	<0.001
LD1	−0.158	0.033	−4.002	<0.001
LD2	−0.411	0.077	−4.836	<0.001
*HIF3A* (λ = 0.001; *R*^2^ = 0.238)				
(Intercept)	6.496	0.619	10.486	<0.001
LD1	−0.260	0.175	−1.484	0.149
LD2	0.513	0.210	2.444	0.021
*EGLN1* (λ = 0.001; *R*^2^ = 0.340)				
(Intercept)	6.004	0.491	12.235	<0.001
LD1	0.479	0.118	4.057	<0.001
LD2	0.318	0.163	1.945	0.059
*EGLN2* (λ = 0.001; *R*^2^ = 0.121)				
(Intercept)	6.691	0.619	10.810	<0.001
LD2	−0.419	0.203	−2.065	0.047

The effects of LD scores from DAPC analyses of each gene on *P*_crit_ were tested with PGLS with lambda = 0.001 (no phylogenetic influence) after model reduction by ANOVA (see Materials and Methods). Lambda values (λ) and *R*^2^ values for each model are given in parentheses.

**Table 4:** Relationships between the physicochemical properties of positively selected amino acid sites in Actinopterygian *HIFA* and *EGLN* and critical oxygen tension (*P*_crit_) at 24 °C, after accounting for phylogeny

Effect	Estimate	SE	*t*	*P*
*HIF1A* (λ = 0.000; *R*^2^ = 0.481)				
(Intercept)	5.577	0.403	13.850	<0.001
LD2	0.084	0.091	0.919	0.364
LD3	0.635	0.113	5.642	<0.001
*HIF2A* (λ = 0.534; *R*^2^ = 0.346)				
(Intercept)	8.130	1.083	7.505	<0.001
LD1	−0.138	0.073	−1.903	0.064
LD2	−0.460	0.122	−3.776	<0.001

The effects of LD scores from DAPC analyses of each gene on *P*_crit_ were tested with PGLS using the model-selected values of lambda (to account for phylogeny) after model reduction by ANOVA (see Materials and Methods). Lambda values (λ) and *R*^2^ values for each model are given in parentheses.

We expected that including phylogeny would reduce the power of sequence variation to explain variation in *P*_crit_ because both the gene sequences and *P*_crit_ (Verberk et al. 2022a) have strong phylogenetic signals. This is not to say that phylogenetically related variation in the physicochemical properties of *HIF3A*, *EGLN1*, and *EGLN2* is unimportant. Rather, it is simply not possible to distinguish the role of adaptation versus shared evolutionary history in shaping this variation. The more striking result was that, even after including the influence of phylogeny, physicochemical variation in positively selected sites of *HIF1A* and *HIF2A* was significantly associated with variation in *P*_crit_ across temperatures.

**Fig. 4.**
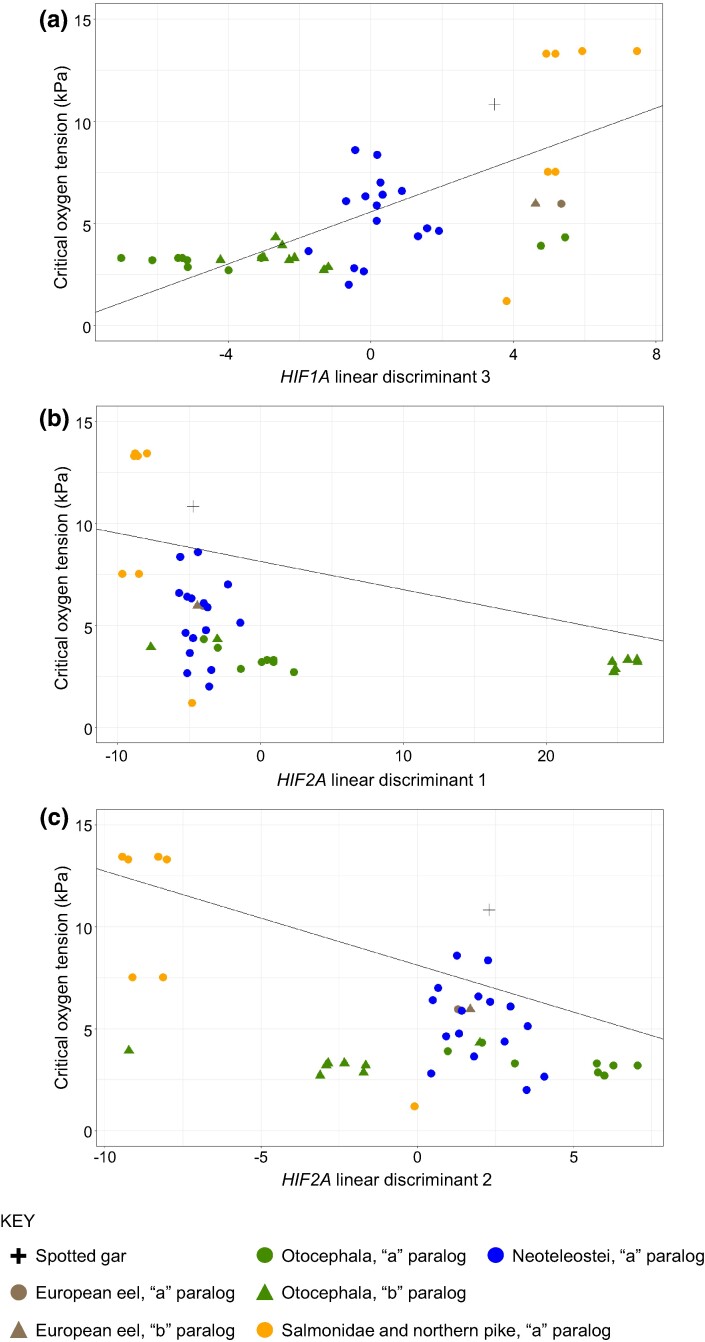
Relationships between physicochemical variation in Actinopterygian *HIF1A* and *HIF2A* and critical oxygen tension (*P*_crit_) at 24 °C. Standardized *P*_crit_ (see Materials and Methods) are plotted against LD scores from DAPC analysis of a) *HIF1A* or b) and c) *HIF2A*. Only LDs having *P* < 0.10 in the final PGLS models are plotted (see Table 4). The regression lines are from PGLS, including the effects of phylogeny. Symbol colors are basal ray-finned fish (spotted gar), black; basal teleost (European eel), brown; Otocephala, green; Salmonidae and northern pike, orange; and Neoteleostei, blue. Symbol shapes are ancestor of teleost-specific duplicates, cross; teleost-specific “a” paralog, circles; and teleost-specific “b” paralog, triangles.

The relationships between the standardized *P*_crit_ at 24 °C and each LD retained in the phylogenetically informed PGLS models (*P* < 0.1) are shown in Fig. 4 (supplementary fig. S8, Supplementary Material online, for *P*_crit_ at 15 °C; supplementary fig. S9, Supplementary Material online, for *P*_crit_ at 28 °C). Variation in *HIF1A* LD3 tended to discriminate between Otocephala, characterized by low *P*_crit_ (high hypoxia tolerance; green symbols), and Salmonidae, characterized by high *P*_crit_ (low hypoxia tolerance; orange symbols) (Fig. 4a; supplementary figs. S8a and S9a, Supplementary Material online). Variation in *HIF2A* LD1 separated Otocephala *HIF2Ab*, specifically those from cyprinids, from *HIF2Aa* in all other fishes (Fig. 4b; supplementary figs. S8b and S9b, Supplementary Material online). Variation in *HIF2A* LD2 tended to segregate salmonid *HIF2Aa* from all others (Fig. 4c; supplementary figs. S8c and S9c, Supplementary Material online). While all of these relationships are significant, or nearly so (e.g. *P* = 0.064 for *HIF2A* LD1 at 24 °C), it is important to note that these LDs explain vastly different amounts of the overall physicochemical variation in the respective genes (Table 2). *HIF2A* LD1 explains ∼80% of the variation in *HIF2A*, whereas *HIF2A* LD2 and *HIF1A* LD3 each explain 14% of the physicochemical variation in those genes.

We carried out a separate analysis using randomly selected amino acid sites to test whether the patterns observed above were due to chance. For this analysis, we selected 52 amino acid sites from *HIF2A* that were not shown to be under positive selection (compared to 53 amino acids found to be under positive selection for this gene, Table 1). DAPC returned four DAPC groups distinguished from one another by three LDs (the same number of groups and LDs as for positively selected sites from *HIF2A*). PGLS without considering phylogeny (λ = 0.001) showed that LD2 was related to standardized *P*_crit_ at all three temperatures (*P* < 0.1; supplementary table S13, Supplementary Material online), although the PGLS models themselves had low *R*^2^ and were not significant. When phylogeny was included in the analysis (model-selected λ), no individual LDs were significantly related to *P*_crit_ at any temperature (supplementary table S14, Supplementary Material online), and correspondingly, no models were significant. These results suggest that the physicochemical properties of randomly selected amino acid sites can be weakly related to variation in *P*_crit_, but that this variation is completely explained by phylogeny. Therefore, the relationships between physicochemical variation at positively selected sites in *HIF1A* and *HIF2A* and *P*_crit_ that were found with PGLS after accounting for phylogeny (Table 4) cannot be explained by random chance.

### Variation in *P*_crit_ Is Best Explained by Physicochemical Variation in *HIF2A*

PGLS models including *HIF2A* LD1 and LD2 explained between 20% and 39% of the variation in *P*_crit_, after accounting for phylogeny (model *R*^2^ from Table 4 and supplementary tables S11 and S12, Supplementary Material online). In addition, LD1 and LD2 together accounted for 94% of the physicochemical variation in amino acid sites putatively under positive selection in this sample of ray-finned fish *HIF2A* (Table 2). These results strongly suggest that sequence variation in *HIF2A* is associated with hypoxia tolerance of fishes. As mentioned above, positive selection in *HIF2A* has been proposed to contribute to the adaptation of various vertebrate groups to high-altitude hypoxia (Beall et al. 2010; Guan et al. 2014; Li et al. 2014; Wang et al. 2015a, b; Eichstaedt et al. 2017; Schweizer et al. 2019; Pamenter et al. 2020). In Tibetan human populations, sequence variation in *HIF2A* is associated with a diminished increase in hematocrit at altitude and lower incidence of chronic mountain sickness (Beall et al. 2010; Basang et al. 2015). In North American deer mice (*Peromyscus maniculatus*), a high-altitude variant of *HIF2A* (*Epas1*) is associated with higher heart rate under hypoxia and potentially blunted adrenal catecholamine release (Schweizer et al. 2019).

In the current study, the first major axis of variation (LD1) distinguished *HIF2Ab* from the Cyprinidae, having high hypoxia tolerance, from all other *HIF2A*, and the second major axis (LD2) separated *HIF2Aa* from Salmonidae, having low hypoxia tolerance, from other *HIF2A*. Recently, Zhao et al. (2023) showed that SNP variation in *HIF2Ab* is correlated with hypoxia tolerance in the blunt nose bream (*Megalobrama amblycephala*), a member of the Cyprinidae. In ray-finned fishes, *HIF2A* is primarily expressed in gill tissue (Townley et al. 2022), where it may be involved in oxygen sensing (Pan et al. 2022). Furthermore, carp and goldfish exhibit gill remodeling during hypoxia or elevated temperature conditions, resulting in greater surface area available for gas exchange (Sollid et al. 2003; Sollid et al. 2005). Whether the variation we document in *HIF2A* contributes to hypoxia tolerance by altering the development, physiology, or morphology of gills in ray-finned fishes remains to be explored.

In comparison to *HIF2A*, PGLS models relating variation in *HIF1A* to *P*_crit_, while explaining a similar amount of variation in *P*_crit_ (Table 4; supplementary tables S11 and S12, Supplementary Material online), retained LD3 which only explained 14% of the physicochemical variation of the positively selected sites in *HIF1A* (Table 2). This result suggests that physicochemical variation in *HIF1A* is a poor predictor of hypoxia tolerance of fishes. This result is consistent with Rytkonen et al. (2007), who failed to demonstrate clear relationships between *HIF1A* amino acid variation and the oxygen sensitivity of a smaller sample of ray-finned fishes. In addition, Mandic et al. (2020) demonstrated that deletion of both paralogs of *HIF1A* (*HIF1Aa/Ab*) in zebrafish had no effect on *P*_crit_ of adult fish. Other metrics of hypoxia tolerance, however, were affected by this double knockout: as larvae, knockout fish displayed higher *P*_crit_ (less hypoxia tolerance) than wild-type larvae, and adult knockout fish became impaired (lost equilibrium) sooner than wild-type adults when exposed to water with oxygen tensions lower than *P*_crit_ (Mandic et al. 2020). Recently, Elbassiouny et al. (2024) documented variation in *HIF1Ab* among knifefish species (order Gymnotiformes, also included in the Otocephala) that was correlated with oxygen levels of their natural habitats. Based upon in vitro assays, these authors proposed that knifefishes from habitats more prone to hypoxia have a variant of *HIF1Ab* capable of greater transactivation of gene expression during conditions of low oxygen. Thus, although our data provide limited support for a relationship between sequence variation in *HIF1A* and *P*_crit_ of adults, there is little doubt that it contributes to other aspects of hypoxia tolerance in fishes.

## Conclusions

The hypoxia tolerance of fishes is a complex organismal phenotype that is almost certainly controlled by several genes (Healy et al. 2018). Despite this complexity, we demonstrate that physicochemical variation in amino acid sites putatively under positive selection in a single gene, the transcription factor *HIF2A*, is strongly associated with variation in the *P*_crit_ across a broad taxonomic range of fishes. The *P*_crit_ of adult fishes is only one measure of how well fishes tolerate reductions in ambient oxygen, albeit the one most widely documented. We cannot exclude the possibility that the variation of other *HIFA* and *EGLN* homologs described here may be related to other metrics of hypoxia tolerance of fishes or linked to variation in other aspects of their life histories. Future studies should explore the functional links between sequence variation in *HIFA* and how fishes, and other aquatic organisms, deal with naturally occurring and human-induced episodes of low dissolved oxygen.

## Materials and Methods

### Gene Identification and Alignment

We used several search terms (including “hif”, “hypoxia”, “hypoxia inducible factor”, “epas”, “endothelial”, “endothelial PAS”, “egl”, “egln”, “prolyl”, and “prolyl hydroxylase”) to identify four *HIFA* and three *EGLN* genes from 28 Actinopterygian species with published genomes at NCBI (https://www.ncbi.nlm.nih.gov) or Ensembl (http://www.ensembl.org/) through March 2023 (supplementary table S1, Supplementary Material online). Full gene sequences were downloaded, and we used BLASTn and exploratory multiple sequence alignments to identify and group sequences with questionable gene descriptions (e.g. “hypoxia-inducible factor 1-like”). We excluded exact duplicate, low quality, or partial sequences from the curated data sets for all genes except for four instances, where partial sequences that had similar gene names or were on the same chromosome encoded sequential exons of a given gene. We suspected these were called partial sequences or were on unplaced scaffolds due to errors in sequencing or annotating. Thus, these sequences were concatenated and used for all analyses (see Gene ID in supplementary table S1, Supplementary Material online, for details). The longest corresponding CDSs were extracted from the full gene sequences, and nucleotide alignments for each gene were conducted in MACSE v2.06 (Ranwez et al. 2011, 2018) with corresponding amino acid alignments being produced with the BLOSUM62 score matrix (Henikoff and Henikoff 1992).

### Phylogenetic Inference

Maximum likelihood (ML) gene trees were inferred for each gene (e.g. *HIF1A* or *EGLN1*) using rapid bootstrapping and subsequent ML search in RAxML v8.2.11 (Stamatakis 2014) as implemented through Geneious Prime v2023.1.2 (www.geneious.com). This was accomplished by drawing bootstrap support values on the best-scoring ML tree from 100 bootstrap inferences using the general time reversible substitution matrix (GAMMA+P-Invar model). All trees were visualized and edited in Geneious Prime v2023.1.2 (www.geneious.com).

### Positive Selection Analyses

We created combined fasta files of the CDS nucleotide alignments and ML trees for each gene to perform selection analyses in the HyPhy package (Kosakovsky Pond et al. 2005; Kosakovsky Pond et al. 2020) through the Datamonkey webserver (Kosakovsky Pond and Frost 2005a; Delport et al. 2010; Weaver et al. 2018). Frameshifts and stop codons that may result from multiple sequence alignments are not allowed in the data used for selection analyses. Therefore, these characters were replaced with the exportAlignment program in MACSE v2.06 (Ranwez et al. 2011, 2018). For each gene and corresponding phylogeny, we used MEME (Murrell et al. 2012) to assess whether individual sites were subject to episodic selection on a proportion of branches and FEL (Kosakovsky Pond and Frost 2005b) to identify sites of pervasive selection. We then implemented BUSTED (Murrell et al. 2015), which evaluates whether a gene has experienced positive selection at any site on at least one branch given a phylogeny. Evidence ratios from BUSTED for sites identified to be under positive selection by MEME and/or FEL were used as additional support for positive selection. The evidence ratio is a log-likelihood ratio that, when >2, provides support for positive selection compared to the null model (Murrell et al. 2015).

MEME provides a robust quantitative improvement compared to traditional models that are unable to detect instances of episodic positive selection due to variable levels of purifying selection pressure across different lineages, resulting in qualitatively different conclusions (Murrell et al. 2012). While FEL evaluates whether some sites evolved primarily under significant purifying selection, MEME can detect the signature of positive selection on certain branches. Unlike traditional models, MEME does not assume constant selective forces across all lineages, which allows the strength and direction of natural selection to vary both from site to site and from branch to branch at a site. This flexibility enables MEME to capture widespread episodic selection, which may have been previously underestimated. However, MEME is limited in that it assumes independent selective pressures between branches, and this assumption might be violated if the ratio of nonsynonymous to synonymous substitutions (ω) changes slowly across a phylogeny (Murrell et al. 2012). Because MEME uses a mixed effects model that allows for different branches and sites to have different ω rates, it is sensitive to updated alignments when sequence data have changed, additional paralogs are included, or species are added to a data set, resulting in a change in ω estimates for some sites or their significance levels, depending on the model fit and phylogenetic signal. It should be noted that even traditional tests of positive selection are sensitive to alignment methods which can lead to different conclusions in detecting positively selected sites (Wong et al. 2008).

### Physicochemical Similarity Assessment

For each *HIFA* and *EGLN* homology group, codons identified as sites under positive selection were scored by five z-descriptors of amino acid physicochemical properties as described by Sandberg et al. (1998). The descriptors included hydrophobicity (z1), steric bulk (z2), polarity (z3), and electronic effects (z4 and z5). When codons were missing due to gaps in the multiple sequence alignments, the column means for z-descriptors were used. Salmonidae and Neoteleostei retain truncated forms of *HIF2Ab* with CDSs less than half the length of “full-length” *HIF2Ab* (Rytkonen et al. 2013; Townley et al. 2022). Preliminary analyses showed that they formed their own physicochemical group (Townley et al. 2022) and they were removed from the analysis of other *HIF2A* because of the large number of missing sites compared to the longer genes. The adegenet package (Jombart 2008; Jombart and Ahmed 2011) in R v4.2.1 (R Development Core Team 2021) was used to summarize all descriptors from all sites by principal components analysis (PCA). DAPC was performed on the minimum number of retained PCs that explained approximately 90% of the variation. DAPC is a robust multivariate method that serves as a bridge between PCA and discriminant analysis (DA). By transforming data using PCA before applying DA, DAPC ensures that the variables under consideration are uncorrelated, which reduces the dimensionality of the data set and ensures that the number of variables is less than the number of analyzed individuals (a necessary condition for DA). DAPC uses the *k*-means algorithm to infer the minimum number of gene groups by clustering PCs to construct linear combinations of the input variables with the greatest variation between groups and the smallest variation within groups. This results in orthogonal LDs that are axes of variation in retained PCs. The amino acids whose physicochemical properties were heavily loaded on each LD (90th percentile) were mapped to associated codons in the multiple sequence alignments for each gene.

### Critical Oxygen Tensions for Target Species

We employed a database of *P*_crit_ values from marine, freshwater, and brackish fishes (Verberk et al. 2022a, b). This database provides multiple *P*_crit_ values for each species and includes metrics describing methodological details associated with each *P*_crit_ estimation, such as the test temperature and test salinity. Additionally, the database encompasses biological factors (e.g. body size, genome size, and metabolic rates) that collectively contribute to the observed variability in *P*_crit_ (Verberk et al. 2022a). A data set of 171 species with a complete set of variables was supplemented with three species for which *HIFA* and *EGLN* sequences were available, but lacking *P*_crit_ values: spotted gar (*L. oculatus*), Atlantic herring (*C. harengus*), and northern pike (*E. lucius*). The first of these represents a lineage of ray-finned fishes lacking the TGD (Braasch et al. 2016), while the latter two represent sister groups of Cyprinidae and Salmonidae, respectively. *P*_crit_ data for these three species were obtained by searching the Web of Science Core Collection using similar keyword combinations as Verberk et al. (2022a). We refit the model of Verberk et al. (2022a) incorporating these three species to estimate standardized values for *P*_crit_ at 15, 24, and 28 °C (corresponding to the 25th percentile, median, and 75th percentile of measurement temperatures included in the database) for the 28 species of ray-finned fishes for which genomes were available. The standardized *P*_crit_ values accounted for the effects of test salinity, test temperature, metabolic rate, genome size, and body mass, as well as the interactions between temperature and genome size and temperature and body mass (Verberk et al. 2022a).

### Relating Hypoxia Tolerance to *HIFA* and *EGLN* Physicochemical Variation

The effects of physicochemical variation at positively selected amino acids in *HIFA* and *EGLN* on standardized *P*_crit_ values were assessed by PGLS models. The scores for each LD were extracted from the DAPC analysis for each member of a gene family (e.g. *HIF1A*) and regressed against standardized *P*_crit_ values at a given temperature (15, 24, or 28 °C). Two PGLS models were conducted for each of the three temperatures using the best-scoring ML gene tree inferred for each gene: a model that does not account for phylogeny (λ = 0.001) and a model corrected for phylogeny by optimizing lambda using ML (λ = ML). Model reduction was performed with analysis of variance (ANOVA) to retain LDs that had *P* < 0.1, and then, PGLS models of the reduced ANOVAs were summarized. A post hoc analysis of 52 random amino acid sites from *HIF2A* was also conducted to compare with the performance and results of the positively selected sites used in the DAPC and PGLS analyses. All methods for this post hoc analysis were the same as those used for the experimental data sets with one exception: PGLS models were not reduced by ANOVA, which would have resulted in null models. Thus, we present results for the full models both without and with phylogenetic correction.

All analyses were conducted in R v4.1.0 (R Development Core Team 2021) by using the following packages: “ape v5.7-1” (Paradis and Schliep 2019), “geiger v2.0.11” (Pennell et al. 2014), “caper v1.0.1” (Orme et al. 2018), “tidyverse v2.0.0” (Wickham et al. 2019), and “phytools v1.5-1” (Revell 2012).

## Supplementary Material

evae183_Supplementary_Data

## Data Availability

Data files and code supporting the analyses, figures, and tables of this study are publicly available on GitHub (https://github.com/felixpleiva/Genetic_basis_Pcrit) and Zenodo (Babin et al. 2023).
