# Supplementary material for: Evolution of Key Oxygen-Sensing Genes Is Associated with Hypoxia Tolerance in Fishes
Source: Genome Biol Evol. 2024 Aug 21;16(9):evae183. doi: 10.1093/gbe/evae183 (PMC11370800; doi:10.1093/gbe/evae183)
Supplement: evae183_Supplementary_Data [file evae183_supplementary_data.zip › Supplemental_Figures_S1_S9.pdf]

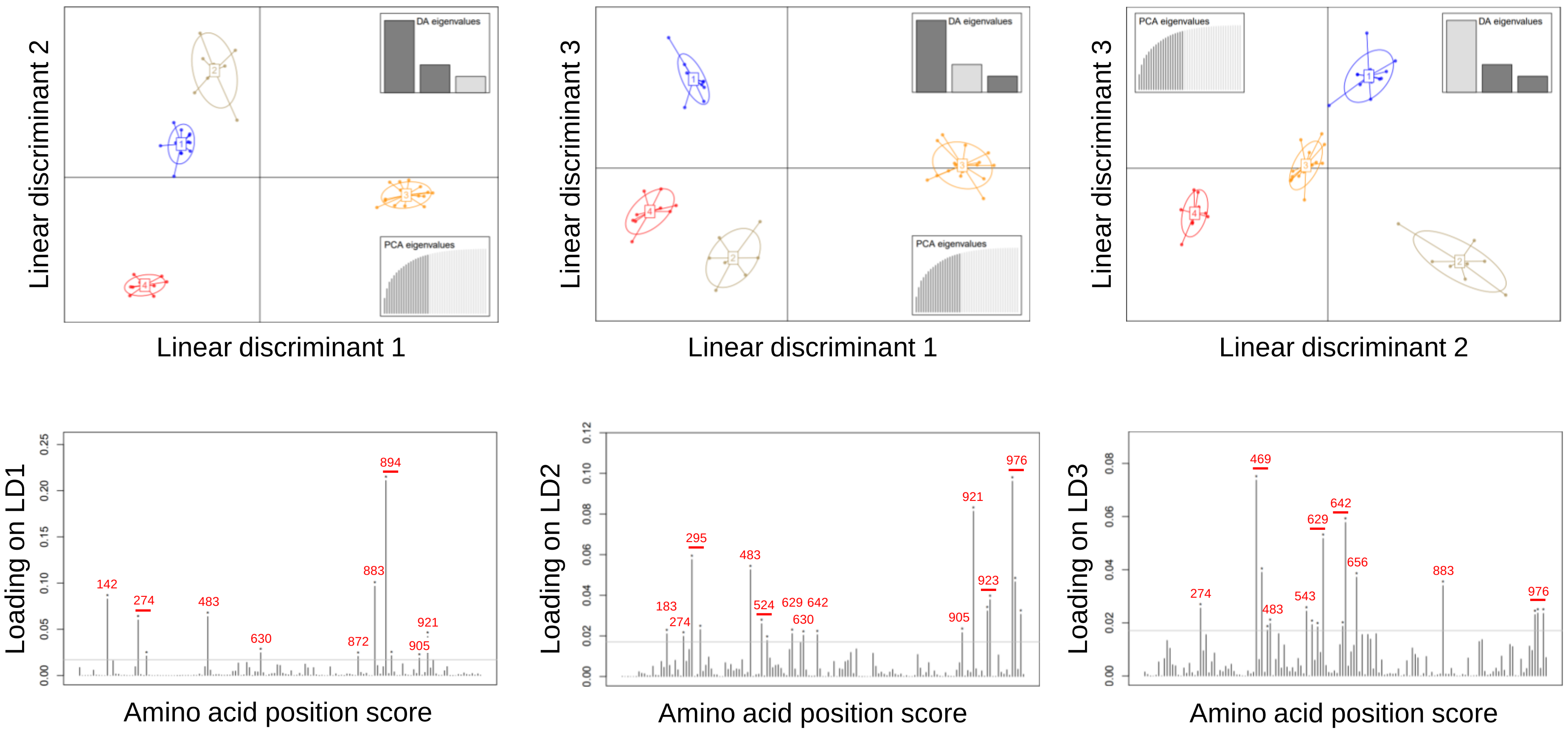

Figure S1. *HIF1A* discriminant analysis of principal components (DAPC) plots (upper row) and corresponding variable loadings (lower row). The first 19 principal components explained 90.8% of the variation in the physicochemical properties of positively selected amino acids of *HIF1A*. Three linear discriminants (DA eigenvalues) distinguished four DAPC groups. DAPC group 1 (blue) included A. anguilla 9087, A. anguilla 9931, C. harengus 1315, C. macropomum 1026, E. lucius 5558, L. oculatus 2513/7742, O. mykiss 5629, O. mykiss 5944, O. tshawytscha 1708, O. tshawytscha 6768, S. salar 0969, S. salar 8919. DAPC group 2 (light brown) included C. auratus 2317, C. auratus 4817, C. auratus 6095, C. auratus 8324, C. carpio 2424, C. carpio 8386, D. rerio 7150, C. idella 5290. DAPC group 3 (orange) included A. polyacanthus 1322, B. splendens 8167, C. lumpus 1277, C. variegatus 7575, F. heteroclitus 7646, G. aculeatus 4896, G. morhua 3958, G. multiradiatus 5803, L. calcarifer 2479, M. salmoides 0761, M. saxatilis 7121, O. niloticus 3737, P. fluviatilis 9074, P. latipinna 9010/3702, S. maximus 6010, T. maccoyii 1349. DAPC group 4 (red) included C. auratus 1035, C. auratus 3370, C. auratus 3374, C. carpio 0693, C. carpio 1721, C. harengus 7766, C. idella 2086, C. macropomum 5526, D. rerio 3292 . Variable loading plots show amino acid position scores in the 90th percentile (indicated by *) of loadings on the respective linear discriminants (LD), with the MSA codon number listed in red above. A red bar indicates the same MSA codon for successive variables.

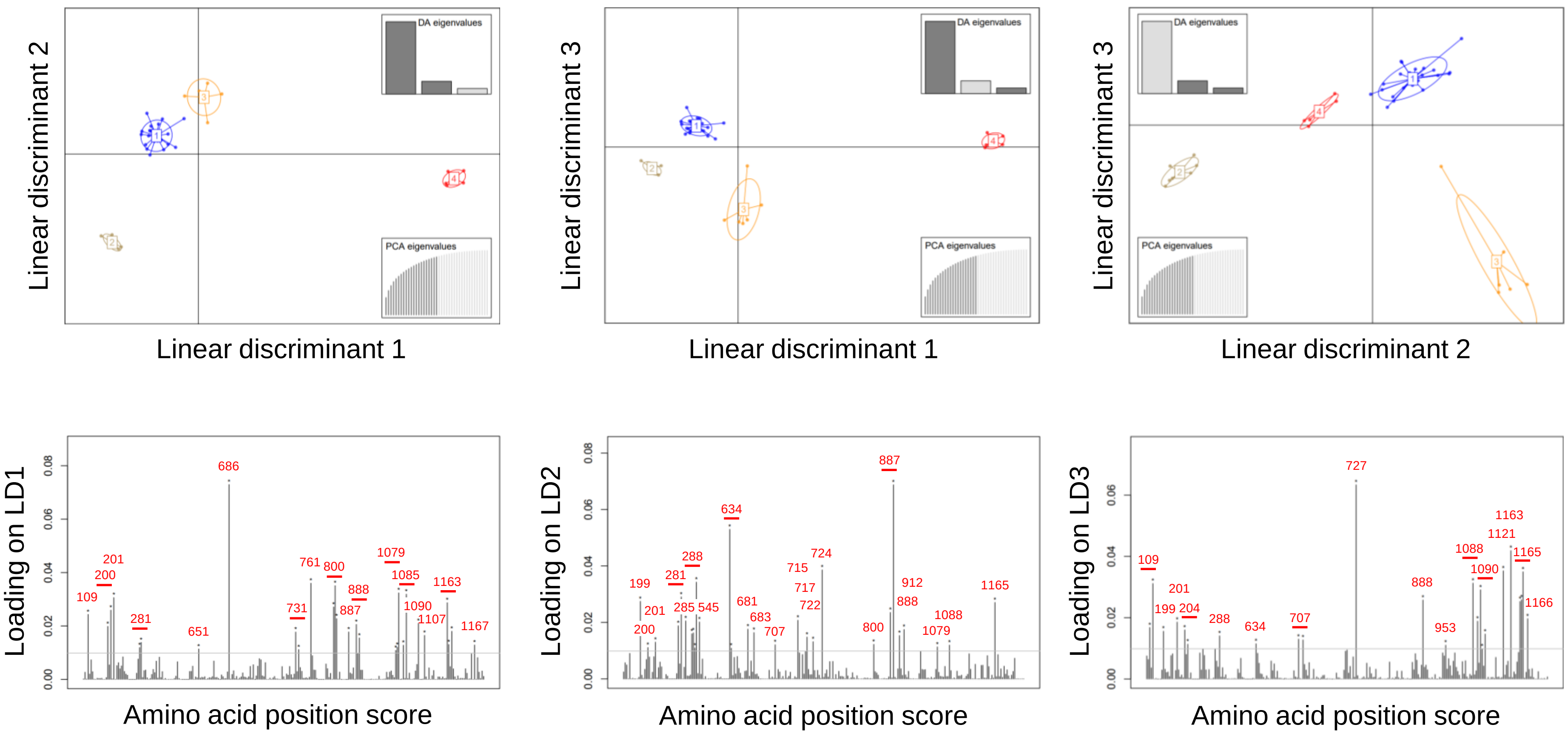

Figure S2. *HIF2A* discriminant analysis of principal components (DAPC) plots (upper row) and corresponding variable loadings (lower row). The first 21 principal components explained 90% of the variation in the physicochemical properties of positively selected amino acids of *HIF2A*. Three linear discriminants (DA eigenvalues) distinguished four DAPC groups. DAPC group 1 (blue) included A. anguilla 0997, A. anguilla 7976, A. polyacanthus 7545, B. splendens 0734, C. harengus 0051, C. lumpus 3823, C. macropomum 0225, C. macropomum 9265, C. variegatus 0627, E. lucius 1253, F. heteroclitus 3321, G. aculeatus 1023, G. morhua 9556, G. multiradiatus 8839, L. calcarifer 2873, L. oculatus 4568, M. salmoides 2382, M. saxatilis 9467, O. niloticus 1768, P. fluviatilis 8035, P. latipinna 5051/2313, S. maximus 3544, T. maccoyii 1414. DAPC group 2 (light brown) included C. harengus 4118, O. mykiss 3001, O. mykiss 9310, O. tshawytscha 0055, O. tshawytscha 4583, S. salar 1028, S. salar 9722. DAPC group 3 (orange) included C. auratus 3130, C. auratus 7223, C. carpio 3442, C. carpio 9786, D. rerio 5192, C. idella 4725. DAPC group 4 (red) included C. auratus 1552, C. auratus 6146, C. carpio 0164, C. carpio 9593, C. idella 3767, D. rerio 6886. Variable loading plots show amino acid position scores in the 90th percentile (indicated by *) of loadings on the respective linear discriminants (LD), with the MSA codon number listed in red above. A red bar indicates the same MSA codon for successive variables.

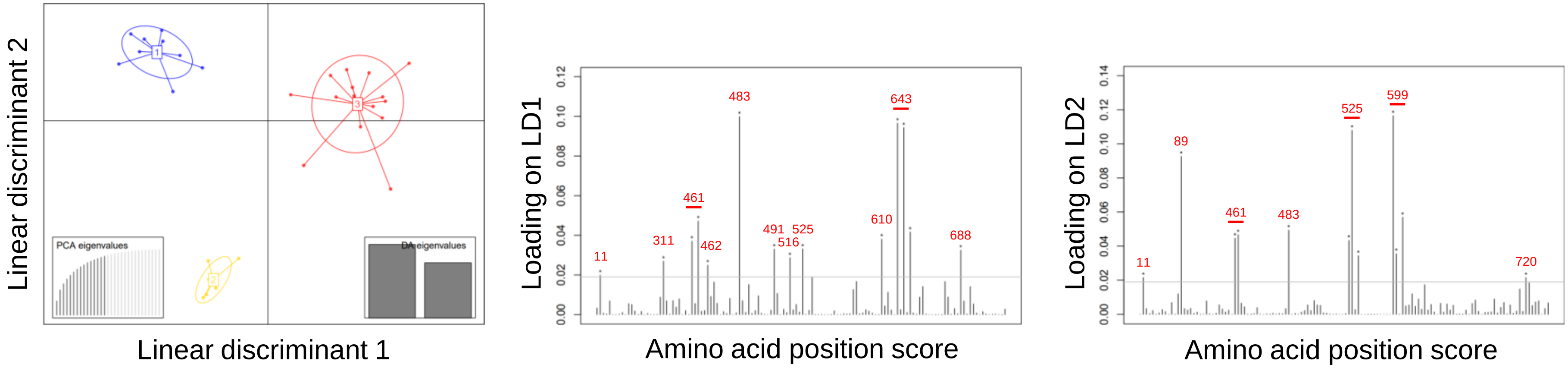

Figure S3. *HIF3A* discriminant analysis of principal components (DAPC) plot (left) and corresponding variable loadings (center and right). The first 15 principal components explained 90% of the variation in the physicochemical properties of positively selected amino acids of *HIF3A*. Two linear discriminants (DA eigenvalues) distinguished three DAPC groups. DAPC group 1 (blue) included A. polyacanthus 3874, C. harengus 9740, C. macropomim 1580, E. lucius 24786, G. morhua 0760, O. mykiss 3712, O. mykiss 7593, O. tshawytscha 5295, S. salar 2598, S. salar 4993. DAPC group 2 (yellow) included C. auratus 5010, C. auratus 8496, C. carpio 3773, C. carpio 4573, C. idella 5103, D. rerio 3376. DAPC group 3 (red) included A. anguilla 9396, B. splendens 7485, C. lumpus 7996, C. variegatus 2506, F. heteroclitus 0192, G. aculeatus 8000, G. multiradiatus 3782, L. calcarifer 2689, L. oculatus 9100, M. salmoides 2851, M. saxatilis 1347, O. niloticus 8832, P. fluviatilis 3479, P. latipinna 6876, S. maximus 9740, T. maccoyii 8449. Variable loading plots show amino acid position scores in the 90th percentile (indicated by *) of loadings on the respective linear discriminants (LD), with the MSA codon number listed in red above. A red bar indicates the same MSA codon for successive variables.

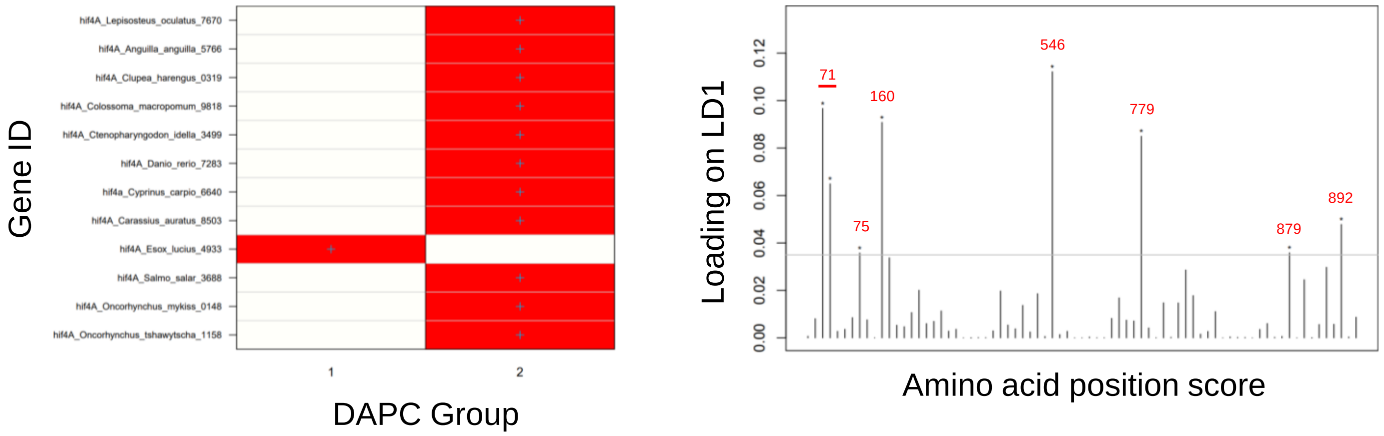

Figure S4. *HIF4A* assignment plot (left) and variable loadings (right). The first seven principal components explained 91% of the variation the physicochemical properties of positively selected amino acids of *HIF4A*. One linear discriminant distinguished two DAPC groups. DAPC group 1 included E. lucius 4933. DAPC group 2 included A. anguilla 5766, C. auratus 8503, C. carpio 6640, C. harengus 0319, C. idella 3499, C. macropomum 9818, D. rerio 7283, L. oculatus 7670, O. mykiss 0148, O. tshawytscha 1158, S. salar 3688. The variable loading plot shows amino acid position scores in the 90th percentile (indicated by *) of loadings on the respective linear discriminant (LD), with the MSA codon number listed in red above. A red bar indicates the same MSA codon for successive variables. A plot of the DAPC result is not available due to the clustering of a single taxon in one of only two groups identified for a single LD.

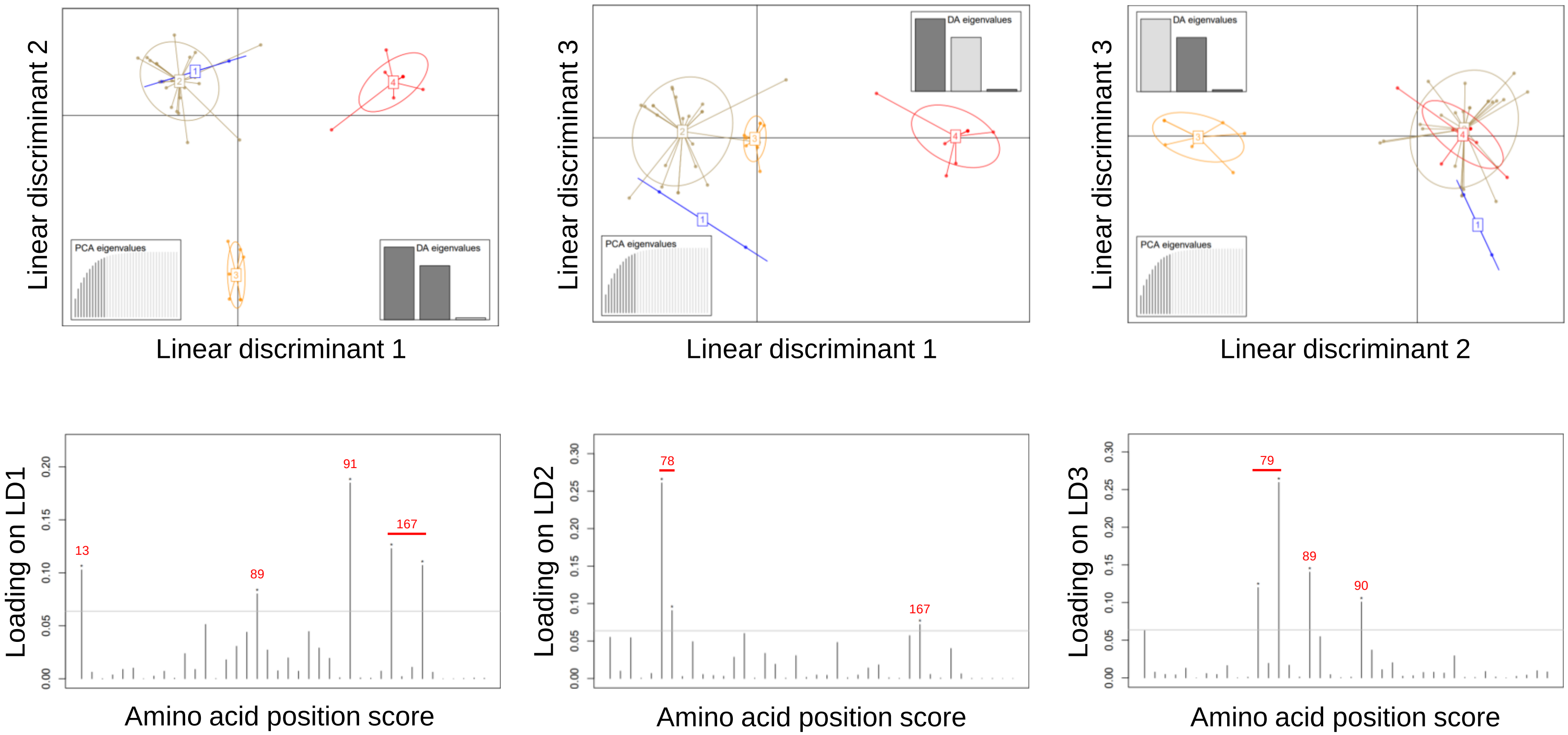

Figure S5. *EGLN1* discriminant analysis of principal components (DAPC) plots (upper row) and corresponding variable loadings (lower row). The first 11 principal components explained 91% of the variation in the physicochemical properties of positively selected amino acids of *EGLN1*. Three linear discriminants (DA eigenvalues) distinguished four DAPC groups. DAPC group 1 (blue) included C. carpio 5546, C. harengus 0941. DAPC group 2 (light brown) included A. polyacanthus 9308, B. splendens 0422, C. auratus 2350, C. auratus 6581, C. carpio 9443, C. carpio 9512, C. idella 4812, C. lumpus 3686, C. variegatus 7820, D. rerio 9385, F. heteroclitus 1348/0184, G. aculeatus 1121, G. morhua 9472, G. multiradiatus 8848, L. calcarifer 5792, M. salmoides 7249, M. saxatilis 9161, O. mykiss 1214, O. niloticus 6862, O. tshawytscha 2094, P. fluviatilis 7839, P. latipinna 7736, S. maximus 3363, S. salar 8747, T. maccoyii 1342. DAPC group 3 (orange) included A. anguilla 7600, C. auratus 1041, C. auratus 5383, C. carpio 3801, C. idella 2502, C. macropomum 0076, D. rerio 6868, E. lucius 6344. DAPC group 4 (red) included A. anguilla 0609, C. harengus 3867, E. lucius 2318, L. oculatus 6669, O. mykiss 6061, O. mykiss 8917, O. tshawytscha 5558, O. tshawytscha 5624, S. salar 4814, S. salar 5313. Variable loading plots show amino acid position scores in the 90th percentile (indicated by *) of loadings on the respective linear discriminants (LD), with the MSA codon number listed in red above. A red bar indicates the same MSA codon for successive variables.

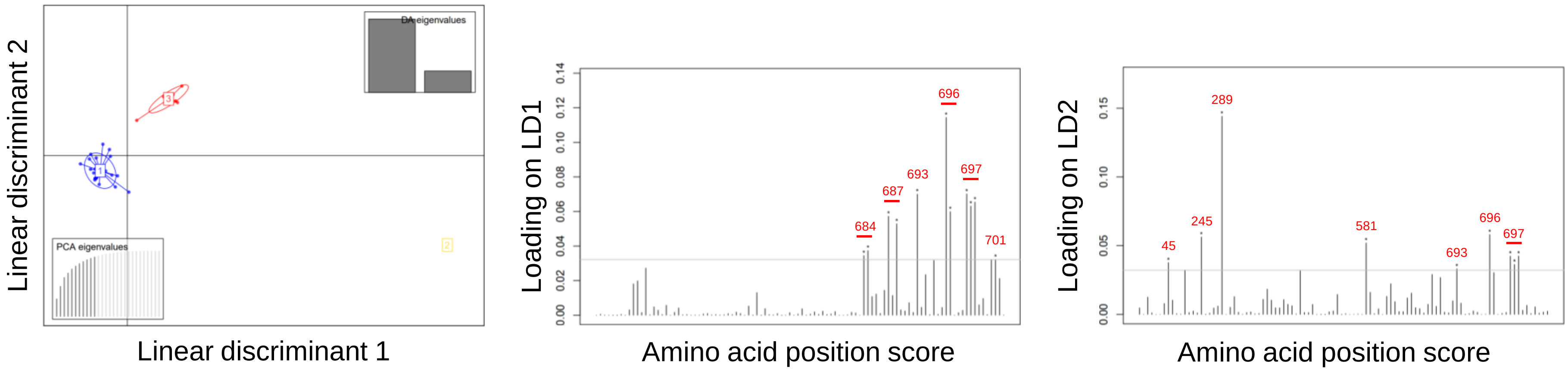

Figure S6. *EGLN2* discriminant analysis of principal components (DAPC) plot (left) and corresponding variable loadings (center and right). The first 11 principal components explained 91% of the variation in the physicochemical properties of positively selected amino acids of *EGLN2*. Two linear discriminants (DA eigenvalues) distinguished three DAPC groups. DAPC group 1 (blue) included A. anguilla 9826, A. polyacanthus 7467, B. splendens 7698, C. lumpus 1644, C. variegatus 4171, E. lucius 2182, F. heteroclitus 6554, G. aculeatus 0907, G. morhua 8963, G. multiradiatus 3940, L. calcarifer 0822, L. oculatus 0383, M. salmoides 2224, M. saxatilis 2165, O. mykiss 3496, O. mykiss 7058, O. niloticus 1117, O. tshawytscha 5089, P. fluviatilis 3539, P. latipinna 3845, S. maximus 9216, S. salar 6807, S. salar 9256, T. maccoyii 8827. DAPC group 2 (yellow) included O. tshawytscha 6681. DAPC group 3 (red) included C. auratus 5280, C. auratus 8594, C. carpio 2858, C. carpio 4525, C. harengus 1526, C. idella 5312, C. macropomum 3212, D. rerio 9569. Variable loading plots show amino acid position scores in the 90th percentile (indicated by *) of loadings on the respective linear discriminants (LD), with the MSA codon number listed in red above. A red bar indicates the same MSA codon for successive variables.

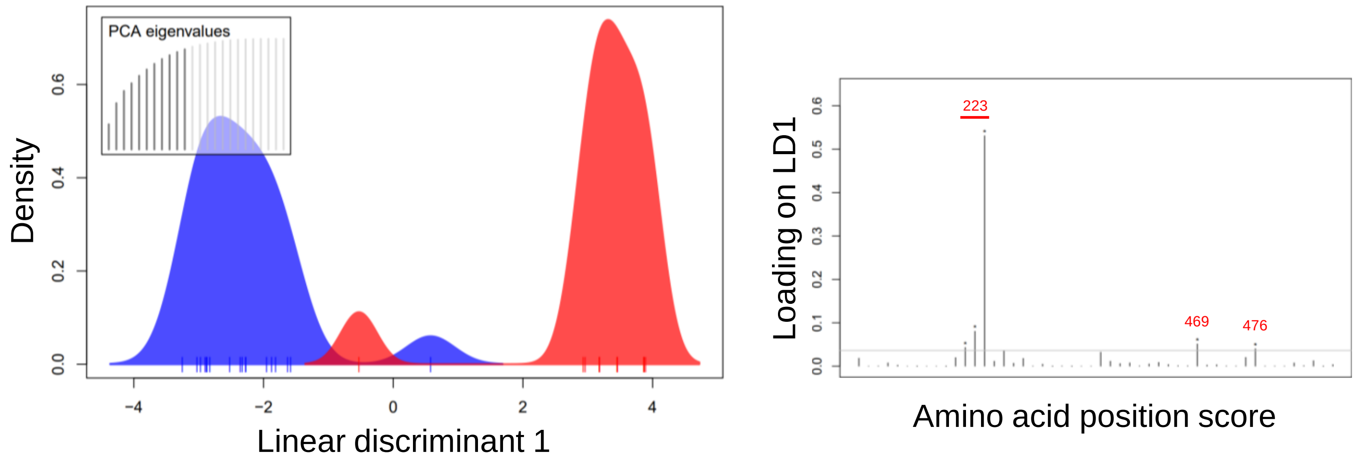

Figure S7. *EGLN3* discriminant analysis of principal components (DAPC) density plot (left) and corresponding variable loadings (right). The first 11 principal components explained 91% of the variation in the physicochemical properties of positively selected amino acids of *EGLN3*. One linear discriminant distinguished two DAPC groups. DAPC group 1 (blue) included A. polyacanthus 9938, B. splendens 7957, C. carpio 8049, C. idella 8723, C. lumpus 1209, C. variegatus 6869, E. lucius 3611, F. heteroclitus 1426, G. multiradiatus 5976, M. salmoides 0676, O. mykiss 7687, O. niloticus 2648, O. tshawytscha 1606, P. fluviatilis 9458, P. latipinna 3955, S. maximus 6054, S. salar 1984, T. maccoyii 1533. DAPC group 2 (red) included A. anguilla 4615, C. auratus 0922, C. auratus 7678, C. carpio 8372, C. harengus 9598, C. macropomum 3274, D. rerio 6602, L. calcarifer 1163, L. oculatus 3659, M. saxatilis 7423, O. mykiss 5778, O. tshawytscha 6840, S. salar 1226. Variable loading plot shows amino acid position scores in the 90th percentile (indicated by *) of loadings on the respective linear discriminant (LD), with the MSA codon number listed in red above. A red bar indicates the same MSA codon for successive variables.

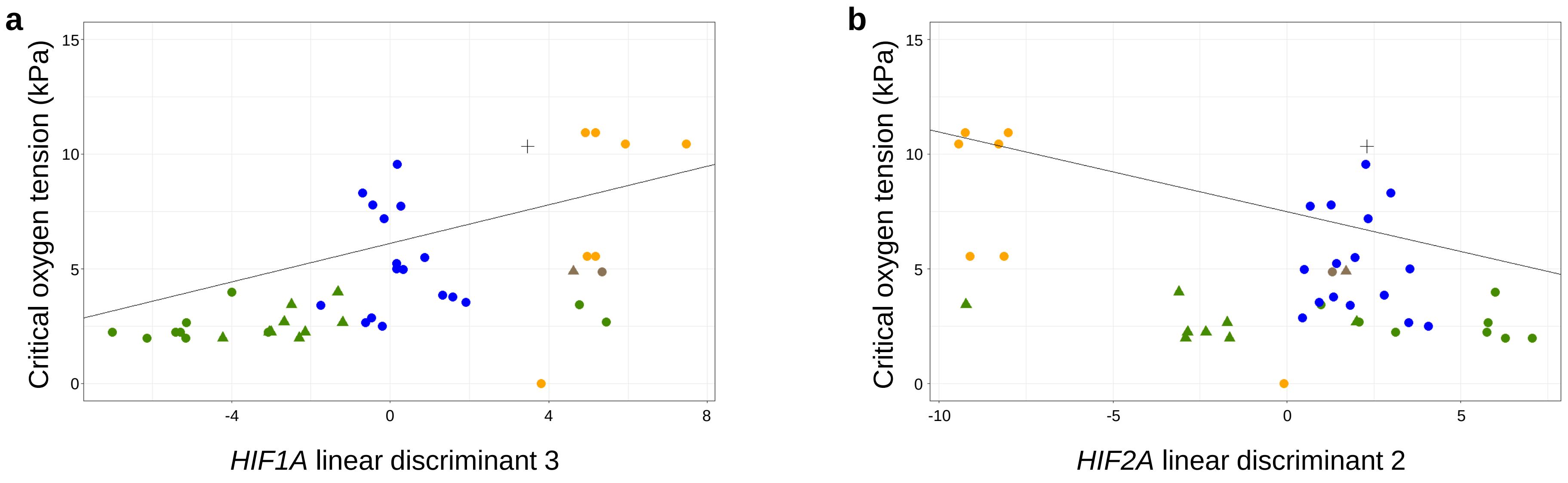

Figure S8. Relationships between physicochemical variation in Actinopterygian *HIF1A* and *HIF2A* and critical oxygen tension (P_{crit}) at 15°C. Standardized P_{crit} (see Methods) are plotted against linear discriminant scores from DAPC analysis of *HIF1A* (a) or *HIF2A* (b). Only linear discriminants having $p < 0.10$ in the final PGLS models are plotted (see Table S11). The regression lines are from PGLS, including the effects of phylogeny. Symbols colors are basal ray-finned fish (spotted gar), black; basal teleost (European eel), brown; Otocephala, green; Salmonidae and northern pike, orange; Neoteleostei, blue. Symbol shapes are ancestor of teleost-specific duplicates, cross; teleost-specific “a” paralog, circles; teleost-specific “b” paralog, triangles.

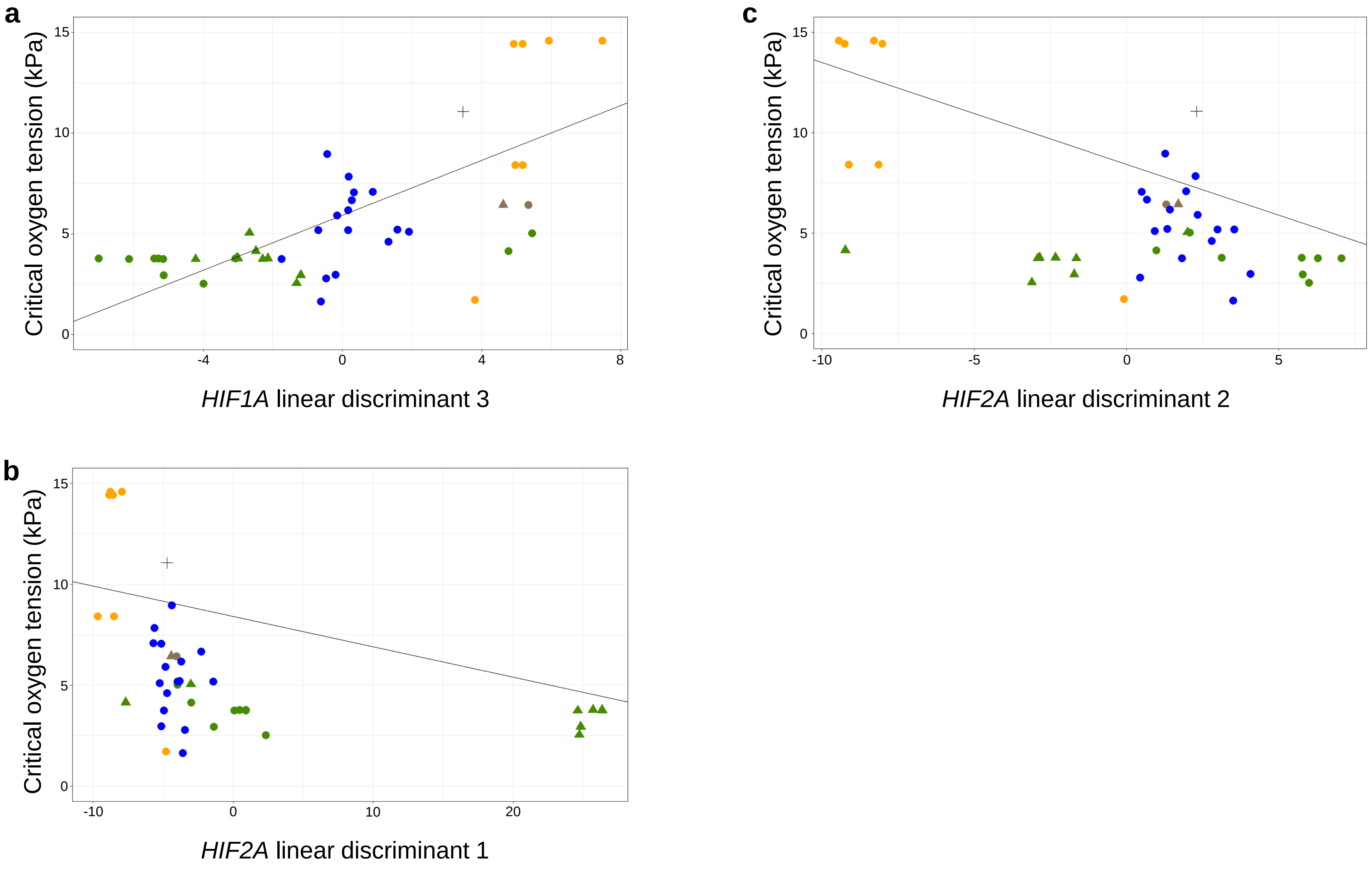

Figure S9. Relationships between physicochemical variation in Actinopterygian *HIF1A* and *HIF2A* and critical oxygen tension (P_{crit}) at 28°C. Standardized P_{crit} (see Methods) are plotted against linear discriminant scores from DAPC analysis of *HIF1A* (a) or *HIF2A* (b, c). Only linear discriminants having $p < 0.10$ in the final PGLS models are plotted (see Table S12). The regression lines are from PGLS, including the effects of phylogeny. Symbols colors are basal ray-finned fish (spotted gar), black; basal teleost (European eel), brown; Otocephala, green; Salmonidae and northern pike, orange; Neoteleostei, blue. Symbol shapes are ancestor of teleost-specific duplicates, cross; teleost-specific "a" paralog, circles; teleost-specific "b" paralog, triangles.
