# Supplementary material for: Evolution of Key Oxygen-Sensing Genes Is Associated with Hypoxia Tolerance in Fishes
Source: Genome Biol Evol. 2024 Aug 21;16(9):evae183. doi: 10.1093/gbe/evae183 (PMC11370800; doi:10.1093/gbe/evae183)
Supplement: evae183_Supplementary_Data [file evae183_supplementary_data.zip › Supplemental_Tables_S9_S14.docx]

**Table S9.** Relationships between the physicochemical properties of positively selected amino acid sites in Actinopterygian HIFA and EGLN and critical oxygen tension (P_crit_) at 15°C, without accounting for phylogeny. The effects of linear discriminant (LD) scores from DAPC analyses of each gene on P_crit_ were tested with Phylogenetic Generalized Least Squares with lambda = 0.001 (no phylogenetic influence) after model reduction by Analysis of Variance (see Methods). Lambda values (λ) and R^2^ values for each model are given in parentheses.

| Effect | Estimate | SE | t | p |
| --- | --- | --- | --- | --- |
| *HIF1A* (λ = 0.001; R^2^ = 0.381) |  |  |  |  |
| (Intercept) | 4.810 | 0.403 | 11.947 | < 0.001 |
| LD2 | 0.056 | 0.090 | 0.621 | 0.538 |
| LD3 | 0.518 | 0.112 | 4.644 | < 0.001 |
| *HIF2A* (λ = 0.001; R^2^ = 0.350) |  |  |  |  |
| (Intercept) | 5.139 | 0.420 | 12.223 | < 0.001 |
| LD1 | -0.139 | 0.044 | -3.135 | 0.003 |
| LD2 | -0.271 | 0.088 | -3.070 | 0.004 |
| *HIF3A* (λ = 0.001; R^2^ = 0.143) |  |  |  |  |
| (Intercept) | 5.734 | 0.567 | 10.106 | < 0.001 |
| LD2 | 0.429 | 0.192 | 2.238 | 0.033 |
| *EGLN1* (λ = 0.001; R^2^ = 0.278) |  |  |  |  |
| (Intercept) | 5.205 | 0.452 | 11.513 | < 0.001 |
| LD1 | 0.349 | 0.109 | 3.202 | 0.003 |
| LD2 | 0.328 | 0.151 | 2.179 | 0.035 |
| *EGLN2* (λ = 0.001; R^2^ = 0.126) |  |  |  |  |
| (Intercept) | 5.835 | 0.552 | 10.564 | < 0.001 |
| LD2 | -0.383 | 0.181 | -2.117 | 0.042 |

**Table S10.** Relationships between the physicochemical properties of positively selected amino acid sites in Actinopterygian HIFA and EGLN and critical oxygen tension (P_crit_) at 28°C, without accounting for phylogeny. The effects of linear discriminant (LD) scores from DAPC analyses of each gene on P_crit_ were tested with Phylogenetic Generalized Least Squares with lambda = 0.001 (no phylogenetic influence) after model reduction by Analysis of Variance (see Methods). Lambda values (λ) and R^2^ values for each model are given in parentheses.

| Effect | Estimate | SE | t | p |
| --- | --- | --- | --- | --- |
| *HIF1A* (λ = 0.001; R^2^ = 0.497) |  |  |  |  |
| (Intercept) | 5.939 | 0.424 | 14.024 | < 0.001 |
| LD2 | 0.095 | 0.095 | 1.001 | 0.323 |
| LD3 | 0.680 | 0.117 | 5.795 | < 0.001 |
| *HIF2A* (λ = 0.001; R^2^ = 0.548) |  |  |  |  |
| (Intercept) | 6.331 | 0.413 | 15.347 | < 0.001 |
| LD1 | -0.164 | 0.044 | -3.776 | < 0.001 |
| LD2 | -0.469 | 0.087 | -5.406 | < 0.001 |
| *HIF3A* (λ = 0.001; R^2^ = 0.271) |  |  |  |  |
| (Intercept) | 6.850 | 0.649 | 10.560 | < 0.001 |
| LD1 | -0.342 | 0.183 | -1.867 | 0.072 |
| LD2 | 0.546 | 0.220 | 2.482 | 0.019 |
| *EGLN1* (λ = 0.001; R^2^ = 0.352) |  |  |  |  |
| (Intercept) | 6.374 | 0.519 | 12.292 | < 0.001 |
| LD1 | 0.533 | 0.125 | 4.270 | < 0.001 |
| LD2 | 0.311 | 0.173 | 1.800 | 0.079 |
| *EGLN2* (λ = 0.001; R^2^ = 0.114) |  |  |  |  |
| (Intercept) | 7.080 | 0.661 | 10.710 | < 0.001 |
| LD2 | -0.433 | 0.217 | -1.999 | 0.055 |

**Table S11.** Relationships between the physicochemical properties of positively selected amino acid sites in Actinopterygian HIFA and EGLN and critical oxygen tension (P_crit_) at 15°C, after accounting for phylogeny. The effects of linear discriminant (LD) scores from DAPC analyses of each gene on P_crit_ were tested with Phylogenetic Generalized Least Squares using the model-selected values of lambda (to account for phylogenety) after model reduction by Analysis of Variance (see Methods). Lambda values (λ) and R^2^ values for each model are given in parentheses.

| Effect | Estimate | SE | t | p |
| --- | --- | --- | --- | --- |
| *HIF1A* (λ = 0.514; R^2^ = 0.148) |  |  |  |  |
| (Intercept) | 6.129 | 1.159 | 5.287 | < 0.001 |
| LD3 | 0.420 | 0.154 | 2.737 | 0.009 |
| *HIF2A* (λ = 0.613; R^2^ = 0.201) |  |  |  |  |
| (Intercept) | 7.499 | 1.201 | 6.244 | < 0.001 |
| LD1 | -0.101 | 0.080 | -1.268 | 0.213 |
| LD2 | -0.348 | 0.132 | -2.629 | 0.012 |

**Table S12.** Relationships between the physicochemical properties of positively selected amino acid sites in Actinopterygian HIFA and EGLN and critical oxygen tension (P_crit_) at 28°C, after accounting for phylogeny. The effects of linear discriminant (LD) scores from DAPC analyses of each gene on P_crit_ were tested with Phylogenetic Generalized Least Squares using the model-selected values of lambda (to account for phylogenety) after model reduction by Analysis of Variance (see Methods). Lambda values (λ) and R^2^ values for each model are given in parentheses.

| Effect | Estimate | SE | t | p |
| --- | --- | --- | --- | --- |
| *HIF1A* (λ = 0.000; R^2^ = 0.497) |  |  |  |  |
| (Intercept) | 5.936 | 0.420 | 14.151 | < 0.001 |
| LD2 | 0.095 | 0.095 | 1.002 | 0.322 |
| LD3 | 0.681 | 0.117 | 5.805 | < 0.001 |
| *HIF2A* (λ = 0.499; R^2^ = 0.392) |  |  |  |  |
| (Intercept) | 8.422 | 1.068 | 7.889 | < 0.001 |
| LD1 | -0.151 | 0.072 | -2.100 | 0.042 |
| LD2 | -0.507 | 0.121 | -4.177 | < 0.001 |

**Table S13.** Relationships between the physicochemical properties of 52 randomly selected amino acid sites in Actinopterygian HIF2A and critical oxygen tension (P_crit_), without accounting for phylogeny. The effects of linear discriminant (LD) scores from DAPC analyses on P_crit_ at three temperatures (15°C, 24°C, and 28°C) were tested with Phylogenetic Generalized Least Squares with lambda = 0.001 (no phylogenetic influence). Lambda values (λ) and R^2^ values for each model are given in parentheses. Full models (shown below) were not statistically significant (p > 0.05).

| Effect | Estimate | SE | t | p |
| --- | --- | --- | --- | --- |
| *HIF2A* – random (P_crit_ at 15°C, λ = 0.001; R^2^ = 0.115) | | | | |
| (Intercept) | 5.135 | 0.502 | 10.229 | < 0.001 |
| LD1 | 0.025 | 0.091 | 0.271 | 0.788 |
| LD2 | -0.210 | 0.096 | -2.201 | 0.034 |
| LD3 | -0.032 | 0.159 | -0.203 | 0.840 |
| *HIF2A* – random (P_crit_ at 24°C, λ = 0.001; R^2^ = 0.105) | | | | |
| (Intercept) | 5.956 | 0.560 | 10.638 | < 0.001 |
| LD1 | 0.032 | 0.101 | 0.313 | 0.756 |
| LD2 | -0.222 | 0.107 | -2.087 | 0.044 |
| LD3 | -0.026 | 0.177 | -0.149 | 0.882 |
| *HIF2A* – random (P_crit_ at 28°C, λ = 0.001; R^2^ = 0.096) | | | | |
| (Intercept) | 6.338 | 0.597 | 10.623 | < 0.001 |
| LD1 | 0.035 | 0.108 | 0.327 | 0.746 |
| LD2 | -0.225 | 0.114 | -1.986 | 0.054 |
| LD3 | -0.022 | 0.189 | -0.118 | 0.907 |

**Table S14.** Relationships between the physicochemical properties of 52 randomly selected amino acid sites in Actinopterygian HIF2A and critical oxygen tension (P_crit_), accounting for phylogeny. The effects of linear discriminant (LD) scores from DAPC analyses on P_crit_ at three temperatures (15°C, 24°C, and 28°C) were tested with Phylogenetic Generalized Least Squares using model-selected values of lambda (to account for phylogeny). Lambda values (λ) and R^2^ values for each model are given in parentheses. Full models (shown below) were not statistically significant (p > 0.05).

| Effect | Estimate | SE | t | p |
| --- | --- | --- | --- | --- |
| *HIF2A* – random (P_crit_ at 15°C, λ = 0.697; R^2^ = 0.023) | | | | |
| (Intercept) | 7.257 | 1.437 | 5.050 | < 0.001 |
| LD1 | 0.032 | 0.098 | 0.326 | 0.747 |
| LD2 | -0.164 | 0.179 | -0.914 | 0.366 |
| LD3 | 0.007 | 0.171 | 0.041 | 0.967 |
| *HIF2A* – random (P_crit_ at 24°C, λ = 0.715; R^2^ = 0.027) | | | | |
| (Intercept) | 8.001 | 1.549 | 5.166 | < 0.001 |
| LD1 | 0.042 | 0.105 | 0.399 | 0.692 |
| LD2 | -0.187 | 0.193 | -0.972 | 0.337 |
| LD3 | -0.004 | 0.183 | -0.023 | 0.982 |
| *HIF2A* – random (P_crit_ at 28°C, λ = 0.723; R^2^ = 0.027) | | | | |
| (Intercept) | 8.332 | 1.636 | 5.094 | < 0.001 |
| LD1 | 0.047 | 0.110 | 0.422 | 0.675 |
| LD2 | -0.198 | 0.203 | -0.975 | 0.336 |
| LD3 | -0.007 | 0.192 | -0.034 | 0.973 |
